# Impact of modality and mode of questioning and testing on memory reports

**DOI:** 10.3389/fcogn.2024.1349511

**Published:** 2024-10-23

**Authors:** Mackenzie R. Riggenbach, Scott D. Gronlund, Phillip R. Zoladz

**Affiliations:** ^1^Department of Psychology, University of Oklahoma, Norman, OK, United States; ^2^Psychology Program, The School of Health and Behavioral Sciences, Ohio Northern University, Ada, OH, United States

**Keywords:** modality of questioning, memory reports, testing effect, written superiority effect, in-person interviewing, virtual interviewing

## Abstract

**Introduction:**

Individuals' memories are assessed in multiple contexts; however, depending on the context, how an individual is questioned may impact the quantity and quality of the details reported. One goal of this study is to investigate how the modality of questioning (individuals talk or write about an event) impacts memory reports. Additionally, being tested on previously learned information improves memory for that information compared to re-studying it. Consequently, another goal is to examine how questioning impacts memory reports compared to a second exposure. We utilized open-ended and pointed questions (true and false).

**Method:**

Participants watched a short video and were questioned (Experiment 1: In-Person; Experiment 2: Virtual) about its contents immediately, 1 week, and 1 month later.

**Results:**

The current study found that writing leads to better quality memory reports than speaking, and the benefit is present 1 week later. Additionally, we found that writing mitigates an anticipated testing benefit, although this depended on whether a pointed or open-ended question was asked. Restudying (vs. immediate testing) led to better performance for the false pointed questions. However, the better performance operated differently depending on whether participants wrote or spoke following restudying, perhaps due to a differential criterion shift between the Restudy-Written and the Restudy-Spoken conditions.

**Discussion:**

We conclude that the impact of the modality of questioning is influenced in several ways by the types of questions asked, which bears significance for many domains because one modality (or a combination) may be more suitable for producing more accurate memory reports as a function of different domains.

## Introduction

Individuals' memories are assessed in multiple contexts. However, depending on the context, individuals may be questioned differently. For example, in the classroom, a student may complete a multiple-choice or essay exam to assess course comprehension; at a crime scene, a police officer might ask a witness to complete an incident report or question a witness about a robbery; in a medical setting, a patient may be asked to list their symptoms on a check-in sheet or report them to a medical professional. Therefore, obtaining reports promoting the most accurate response is important. Thus, one focus of the current study is to examine the impact of a spoken or written report on the number and types of details reported.

Furthermore, in some contexts, individuals' memories may be assessed initially, or they may be provided with an opportunity to reengage with the information beforehand. For example, in the classroom, a student may restudy material before taking an exam or could be given a quiz; at a crime scene, an eyewitness may be questioned once or by several detectives about an incident; in a medical setting, a patient may be re-experiencing symptoms of an illness or experiencing and recounting them for the first time. Therefore, another focus of this study is the impact of reengaging with information compared to initial questioning on the quantity and quality of details reported.

It is also important to consider the dynamic of the memory assessments. For example, in an eyewitness setting, a witness may not predict the interview questions, the structure of the interview, or the kinds of questions they will be asked. However, in a classroom setting, this is not always the case, as students usually know what information they need to study and the type of test they will take. Therefore, the unanticipated nature of the questioning in an eyewitness setting compared to a classroom setting could impact individuals' expectations and, subsequently, the demands placed on their memories. Additionally, a consequence of reengaging with information or having one's memory assessed is that it alters the timeframe over which memories are evaluated and compared. Thus, of additional interest are changes in memory reports across time.

Overall, the goal of the study is to provide support for practical advice on how to improve memory in various real-world scenarios while also assessing potential interactions between variables, as memory can be impacted by more than one at a time. For example, the present study will allow us to gain evidence regarding how to question a witness or how students should prepare for their exams while being sure to consider how re-engaging with information over time and the subsequent passage of time can impact one's ability to do either task successfully.

Prior research reveals two competing ideas regarding the impact of the modality of questioning. Research that supports a written superiority effect suggests that writing is better because it allows for self-pacing and the ability to monitor what information has previously been produced. Sauerland et al. (2014) found that, in general, written free recalls led to better memory performance compared to speaking. Kraus et al. (2017) conducted several types of interviews after participants watched a video of a criminal event. Self-administered interviews (SAI), police officer questioning (POQ), and written free recall (FR) techniques were used for questioning. The SAI, a structured questionnaire that witnesses fill out, led to reports of more correct victim and setting details compared to the participants in the POQ or FR conditions. The SAI group also reported more correct offender and action details compared to the FR group. However, the POQ group did report more offender details compared to both the SAI and FR groups. This study suggests that writing (SAI) leads to better memory performance compared to speaking (POQ), although it is possible that how different written interviews are structured impacts the quality and quantity of eyewitness reports. In contrast, other work suggests that writing places higher demands on working memory because writing is slower than talking, less practiced, and requires activation of grapheme representations for spelling words (Kellogg, 2007).

On the other hand, research that supports a spoken superiority effect suggests that speaking about an event leads to better memory performance because it demands fewer cognitive resources (Kellogg, 2007). In a non-forensic setting, Kellogg (2007) presented a narrative story and found that participants in the spoken condition reported more correct propositions. Sauerland and Sporer (2011) found that having participants talk about a video event led to more detailed and accurate crime descriptions and more accurate central perpetrator details, although writing was better for reporting peripheral perpetrator details. However, it is important to note that although speaking is considered more productive, it is not necessarily more efficient. For example, when speaking, individuals may repeat what they have previously stated. Mechanisms thought to induce a spoken superiority effect include that speaking requires less muscular energy, is acquired earlier in life, and therefore is easier and more practiced (Sauerland and Sporer, 2011). As a result, speaking is thought to lead to a lower level of cognitive demand. Consequently, if cognitive load is low when speaking, individuals may have more working memory capacity available to report and describe details that require more effortful retrieval.

In addition to assessing memory with either a written or spoken report, evaluation can induce a testing effect because initial questioning can function as a test. Roediger and Karpicke (2006) showed that immediately testing individuals after reading a passage led to better long-term retention rates compared to merely restudying the passage. This effect remained even after a retention interval of a week. Additionally, research suggests that rates of forgetting can differ as a function of restudying vs. repeated testing and timing (Wheeler et al., 2003). More specifically, the forgetting of 40 items over an interval of 7 days occurred much faster in the study-only condition compared to the repeatedly tested condition. The ability to learn more from being tested provides an avenue to examine whether the modality of questioning mitigates the testing effect. This is particularly relevant because it is possible that testing and modality could interact, and under some circumstances, there may be instances where modality effects are no longer present or strong under. For example, Rohrer et al. (2005) conducted a study where participants who learned a set of stimulus material perfectly but further engaged in studying (overlearners) recalled more than the low learners on a 1-week test. However, these immediate benefits greatly diminished on the long-term retention tests. These findings suggest that overlearning is an ineffective strategy for learning material for longer periods of time. For our purposes, these findings support the notion that one variable (testing or modality) could dominate the other and subsequently impact memory reports. Therefore, elucidating these differences is crucial to understanding memory mechanisms, especially in different contexts, because it is possible that modality effects may change under optimal (or not) testing scenarios. For practical purposes, it is important to understand how testing and modality may interact.

Parsing out potential differences between a written test compared to a spoken test is important, especially as it relates to the types of details reported. For example, some research suggests that testing can increase the rate of semantically related false memories when there is a theme within a set of stimuli (McDermott, 2006). Thus, investigating the impact of testing and modality of questioning on true and false information is important. In addition, research suggests that different types of information are recalled based on how the information was previously encoded. Fuzzy-Trace Theory (FTT) posits that individuals encode details of an event as a function of gist and verbatim information (Reyna and Brainerd, 1995; Brainerd and Reyna, 2005). According to FTT (Reyna and Brainerd, 1995), studying promotes verbatim processing, whereas testing promotes gist processing (Bouwmeester and Verkoeijen, 2011). Consequently, when a theme is present within a set of stimuli, the enhancement of gist processing associated with testing serves as a helpful retrieval cue. However, restudying may be more effective at promoting retention through the enhancement of verbatim processing (Delaney et al., 2010). Additionally, research investigating memory for repeated events has found that participants in the repeated-event condition were more likely than their counterparts to report general details (Powell and Thomson, 1996) because they can recognize commonalities across exposures of the event (Brainerd et al., 2008). However, Theunissen et al. (2017) found that participants in the repeated-event condition were less accurate than the single-event condition. Therefore, it is important to investigate how repeated exposure impacts memory reports.

Studies that have investigated the modality of questioning have minimally investigated how the passage of time can impact these types of memory reports, and research that does so typically uses a retention interval of a week or shorter. An individual's memory may be assessed at various times following initial encoding, and it is likely that the memory report changes over time as a function of subsequent questioning. Kraus et al. (2017) found that those who completed the SAI immediately after observing the crime reported more correct details without a loss of accuracy 1 week later and had higher accuracy in the Cognitive Interview (CI) (Fisher and Geiselman, 1992) compared to participants in the FR and no-initial interview group. Additionally, Warren and Lane (1995) manipulated the type of initial test (no test, neutral, or misleading) and the type of second test that occurred 1 week later (no test, neutral, or misleading). They found that immediate neutral testing led to an enhancement in inoculating against forgetting and suggestibility. Pansky and Tenenboim (2011) found results consistent with Warren and Lane (1995) at a 48-h delay. It is important to investigate how memory reports change over longer periods in conjunction with a testing effect because Butler and Roediger (2007) found that being immediately tested can improve final recall even 1 month after the initial encoding.

Given the limited and contradictory evidence of the effect of the modality of questioning on memory reports, in conjunction with possible testing effects and question timing, the goal of the present study is to gain a greater understanding of how modality, testing effects, and timing interact to impact memory reports for correct and incorrect information. Based on the findings from Sauerland et al. (2014), we expect the participants in the written condition to report more correct details compared to participants in the spoken condition. We also expect a written superiority effect when questioned immediately, as evidenced by the findings from Kraus et al. (2017). Additionally, given the robust findings of the testing effect, we anticipate that participants who are tested (either by writing or speaking) during Phase 1 of our experiments will show a testing benefit compared to those that restudy the video. Lastly, we expect participants who restudy the information, instead of being immediately questioned, will endorse false questions to a lesser extent because FTT posits that restudying promotes verbatim processing, which should help participants to identify a false question that asks about an event that did not happen.

## Experiment 1

### Method

#### Participants

A statistical power analysis was conducted using GPower 3.0.10 (Faul et al., 2007) to determine the sample size, which was based on a Cohen's f effect size estimate of 0.35. The analysis recommended that 28 participants be recruited per condition for our design (power = 0.95). A medium-large effect size was chosen to remain consistent with findings from previous memory reporting research. A total of 125 introductory psychology students (29 males, 96 females; *M*_Age_ = 19.04 years, *SD*_Age_ = 2.21) from the University of Oklahoma (*N* = 95) and Ohio Northern University (*N* = 30) participated in this study in exchange for partial course credit resulting in a *post hoc* power of 0.91. All students were enrolled in an introductory psychology course and were recruited via a university recruitment portal (SONA study flier). The flier informed potential participants that they would watch a video and then be asked questions about the video at three different time points. Participants received a maximum of 2.5 research credits for their psychology course. They received credit following the completion of two laboratory sessions and one email response. To participate, students were at least 18 years of age and able to provide consent. In addition, participants indicated that they were proficient in English.

The present study is a 2 (Modality of Questioning: Written vs. Spoken) × 2 (Test vs. Restudy) × 3 (Timing: Immediate, 1-week delay, and 1-month delay, hereafter denoted Phase 1, Phase 2, and Phase 3) incomplete factorial design. The modality of questioning and testing vs. restudying is a between-subjects factor, and participants were randomly assigned to one of the following conditions: Restudy-Written (*n* = 30), Restudy-Spoken (*n* = 33), Written-Written (*n* = 31), and Spoken-Spoken (*n* = 31). Timing is a within-subject factor.

All participants' data were kept anonymous and separate from identifying information. No significant risks were encountered by the participants, and they were treated in accordance with APA (American Psychological Association) ethical standards. The study was approved by both the University of Oklahoma and Ohio Northern University Institutional Review Boards (IRBs - #11236).

### Materials

Participants completed a demographic survey that involved them self-reporting their gender and age. They then viewed an 8-min excerpt from the Disney movie *Looking for Miracles* (Grant and Sullivan, 1989), which depicts the adventures of two brothers at summer camp. This video was chosen because it is an older film and is unlikely to have been previously seen by the participants. After watching the video, all participants were asked whether they had seen the video (*n* = 0). This video also was used because the dynamics of each scene allowed participants to have opportunities to report a multitude of details. Following the video, participants were asked questions about the video at different time points. Both the video and question materials are like those used in previous studies (Zaragoza et al., 2001; Zoladz et al., 2017). Depending on the condition, participants either wrote about what they saw in the video or spoke about it to a research assistant while an audio recording device (iPad) recorded the interaction.

A norming study (*n* = 11) was conducted to determine which reported details would be classified as central or peripheral to the video. These participants watched the video and were asked to report everything that they could remember. Details reported by more than six of the participants were classified as central details and details reported by five or fewer of the participants were classified as peripheral details. On average, a central detail was reported by 7.5 participants and a peripheral detail was reported by 3.7 participants.

Participants were queried with open-ended questions, pointed questions, and a combination of the two, by trained undergraduate research assistants. The open-ended questions were general questions asking about each of the three main scenes in the video. For example, “The first scene took place in the dining hall. Please talk about what events occurred, who was in the scene, describe the people who were there, and any other details that you can remember, such as, did any important conversations happen?” The open-ended questions were asked in storyline order. These questions were framed in a way to serve as guideposts to help participants organize their thoughts and report subsequent details, though they were free and encouraged to recall everything they could remember. If questioning occurred during the first phase of the experiment, only open-ended questions were used.

When questioned during the second and third phases of the experiment, the exact same open-ended questions were used, plus the addition of the pointed questions. The pointed questions used in Phase 2 were identical to those asked in Phase 3. Participants were asked all 13-pointed questions in storyline order, with five false questions interspersed amongst the eight true questions (but still following the order of events in the video). The true pointed questions asked about an event or detail that appeared in the video, whereas the false pointed questions asked about an event or detail that was plausible but did not occur in the video. The false pointed questions allowed us to determine how the susceptibility to false information might change depending on the modality of questioning. Participants were not forced to answer; they could indicate that an event did not happen or that they could not remember an answer. An example of a true question is, “The cook brought out a cake because it was one of the boy's birthdays. What did the cake say?” An example of a false question is, “After Delaney fell, where did he say that he injured himself?” Participants were not forced to answer; they could indicate that an event did not happen or that they could not remember an answer. Participants' responses that included anything other than “that did not happen” when answering a false question were coded as endorsing the false question and, therefore, provided an incorrect answer, unless they indicated that they did not know the correct answer. The amount of questions asked and the ordering of questions is similar to how they were presented in the studies conducted by Zaragoza et al. (2001) and Zoladz et al. (2017).

#### Procedure

**Figure 1:**
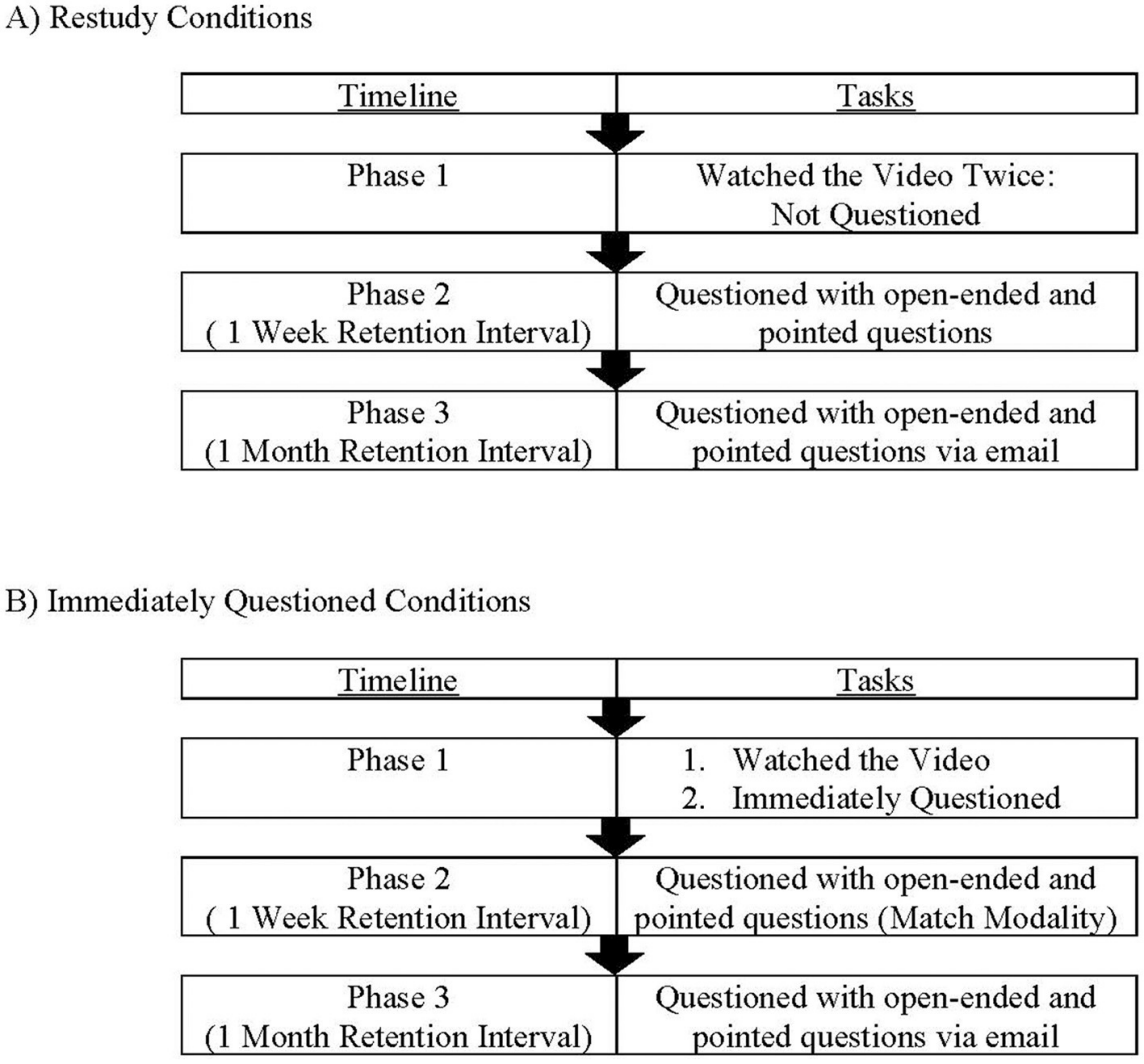
**(A)** Restudy conditions. Restudy-Written (RW) and Restudy-Spoken (RS). **(B)** Immediately questioned conditions. Written-Written (WW) and Spoken-Spoken (SS).

After obtaining informed consent from all participants, participants were asked if they would provide their cell phone numbers to the researcher to receive session reminders throughout their 1-month sequence of sessions. Participants were not required to provide their cell phone numbers. Next, all participants completed the demographic survey. Following the completion of the survey, all participants watched the video. The previously mentioned procedural steps were identical for all participants. It is at this point that the procedure changes depending on the condition. Figure 1A displays the tasks and timeline followed by the participants who were *not* questioned about the video in Phase 1 and instead re-watched the video (Restudy Conditions). Figure 1B depicts the timeline followed and the tasks completed by the participants who were questioned about the video in Phase 1 (Immediately Questioned Conditions).

##### Restudy conditions

After the video ended, participants in the two restudy conditions (Restudy-Written and Restudy-Spoken) watched it again. These participants were not questioned during Phase 1. This phase concluded with a reminder of their next session and then dismissal. The testing effect will be assessed by comparing these participants to those in the Immediately Questioned conditions (described below).

One week later, participants were randomly assigned to write (Restudy-Written) or speak (Restudy-Spoken) during the Phase 2 questioning. Before beginning, participants were asked to indicate the two main characters' names (Delaney and Sullivan). If participants answered incorrectly, the researcher informed them of the correct answers. The purpose of having the participants identify the characters, and be corrected, if necessary, was to make sure that they could correctly reference the two main characters. Participants were then queried with open-ended questions and then with pointed questions. However, depending on the condition, participants either wrote out their responses on lined sheets of paper (Restudy-Written) or spoke to a research assistant while an audio recording device recorded the interaction (Restudy-Spoken). Following the questioning, participants were debriefed. They also were reminded that they would receive an email in 3 weeks to complete Phase 3. One month following Phase 1, participants received an email containing the open-ended and pointed questions along with instructions for how to complete Phase 3. Participants were given 1 week to return their responses. Phase 3 should have taken participants about 15 min to complete.

##### Immediately questioned conditions

Participants randomly assigned to the immediately questioned conditions (Written-Written or Spoken-Spoken) watched the video only once and then were immediately questioned. Participants were first asked the warm-up questions about the main characters' names before the open-ended questions (i.e., no pointed questions were asked). Those in the Written-Written condition wrote their responses to the questions on lined sheets of paper, whereas those in the Spoken-Spoken condition spoke their responses into the audio recorder. Following the questioning, participants were reminded of their next session and dismissed.

One week later, participants were queried using open-ended questions and then pointed questions. The modality of questioning was not mixed. That is, participants in the written conditions (Written-Written) wrote their responses on lined sheets of paper during Phase 2. Participants in the spoken conditions (Spoken-Spoken) for Phase 2 spoke to a research assistant while their responses were recorded with an audio recorder. Following the questioning, participants were debriefed and then reminded that they would receive an email to complete Phase 3 in 3 weeks. The email questioning proceeded as described above. Regardless of condition, all participants typed their responses to the Phase 3 questions.

Note that for these in-person experimental sessions, a researcher was present throughout Phases 1 and 2, regardless of condition type. Multiple research assistants helped with data collection; however, participants had the same research assistant for their Phase 1 and Phase 2 sessions. The researcher set up the video and then sat across from the participant during the entire session to ask all questions and record the spoken responses. All researchers were trained on the proper protocol for questioning participants and were blind to the experimental hypotheses. They responded to each answer with a transitory comment such as, “Okay, the next question is....” This was meant to reduce chances of confirmatory feedback or other cues that might indicate to the participants the verity of their responses (Zaragoza et al., 2001). Researchers also recorded the time it took to complete the questioning, though participants were free to take as much time as they needed. All lab sessions had to be completed sequentially. That is, to participate in Phase 3, Phases 1, and 2 must have been completed within the allotted timeframe.

### Results

A total of 125 participants completed Phase 1. Of those, 118 (94.4% return rate) participants completed Phase 2, and 96 (76.8% response rate) participants completed Phase 3. Only participants who completed at least Phases 1 and 2 were included in the subsequent analyses. However, before conducting any analyses, the data were cleaned, which resulted in an additional 10 participants being removed because of technical errors or not completing Phases 1 and 2. Additionally, the data were examined for normality, and participants' scores for each of the dependent variables were assessed for outliers. Outlying data points were removed; however, only the single data point was removed, not the participant. Therefore, 115 (out of 118) participants' data were used for the Phase 1 and Phase 2 data analyses, and 91 (of 96) participants' data were used for the Phase 3 analyses.

All audio recordings were transcribed before coding. Recordings were coded by three individuals. Interrater reliability scores ranged between a Kappa value of 0.77 and 1.00. All coding disagreements were discussed amongst the coders until a mutual decision could be reached. Open-ended responses were coded for central and peripheral details, intrusions, and any other detail reported correctly but not deemed central or peripheral to the video (based on the norming study). For all analyses, we use partial eta squared as the appropriate effect size estimate. The reasonable effect sizes when interpreting partial eta squared are $\eta_{p}^{2}$ = 0.01, $\eta_{p}^{2}$ = 0.06, and $\eta_{p}^{2}$ = 0.14 for small, medium, and large effects, respectively.

#### Phase 1

To reiterate, participants in the two restudy conditions (Restudy-Written and Restudy-Spoken) were not questioned during Phase 1. In the Written-Written and Spoken-Spoken conditions, the total number of central, peripheral, and correct other details reported during Phase 1 were combined to reflect the overall correct number of details reported for each participant. A one-way ANOVA was conducted to compare the total correct number of details reported in the Spoken-Spoken and Written-Written conditions. Participants in the Written-Written condition (*M* = 35.89, *SD* = 7.40) reported significantly more correct details overall compared to participants in the Spoken-Spoken condition (*M* = 29.25, *SD* = 11.73). We also separately analyzed the number of central and peripheral details reported. Participants in the Written-Written (*M* = 14.37, *SD* = 2.87) condition reported significantly more central details than those in the Spoken-Spoken (*M* = 12.18, *SD* = 3.87) condition. Participants in the Written-Written (*M* = 7.48, *SD* = 2.53) condition also reported more peripheral details than participants in the Spoken-Spoken (*M* = 6.11, *SD* = 2.59) condition. Therefore, the written benefit extends to both the most important details (central) as well as to peripheral details. All *F-tests* and relevant descriptive statistics are reported in Table 1.

**Table 1:** Experiment 1: Phase 1 *F-tests* and relevant descriptive statistics.

**Dependent variable**	**Statistical test**	**Descriptive statistics**	***95% C.I*.**
**Total correct number of detail reported**			
Written vs. Spoken	*F*_(1, 53)_ = 6.247, *p* = 0.016, *$\eta_{p}^{2}$*= 0.105 (power = 0.70)	Written: *M* = 35.89, *SD* = 7.40 Spoken: *M* = 29.25, *SD* = 11.73	Written: 33.93–37.84 Spoken: 26.15–32.35
**Number of central details reported**			
Written vs. Spoken	*F*_(1, 53)_ = 5.659, *p* = 0.021, *$\eta_{p}^{2}$*= 0.096 (power = 0.66)	Written: *M* = 14.37, *SD* = 2.87 Spoken: *M* = 12.18, *SD* = 3.87	Written: 13.61–15.13 Spoken: 11.16–13.20
**Number of peripheral details reported**			
Written vs. Spoken	*F*_(1, 53)_ = 3.961, *p* = 0.052, *$\eta_{p}^{2}$*= 0.07 (power = 0.52)	Written: *M* = 7.48, *SD* = 2.53 Spoken: *M* = 6.11, *SD* = 2.59	Written: 6.81–8.15 Spoken: 5.43–6.79
**IBA**			
Written vs. Spoken	*F*_(1, 53)_ = 4.967, *p* = 0.03, *$\eta_{p}^{2}$* = 0.083 (power = 0.59)	Written: *M* = 0.56, *SD* = 0.12 Spoken: *M* = 0.48, *SD* = 0.16	Written: 0.53 −0.59 Spoken: 0.44 −0.52
**OBA**			
Written vs. Spoken	*F*_(1, 53)_ = 0.041, *p* = 0.840, *$\eta_{p}^{2}$* = 0.001 (power = 0.06)	Written: *M* = 0.90, *SD* = 0.06 Spoken: *M* = 0.90, *SD* = 0.07	Written: 0.88 −0.92 Spoken: 0.88 −0.22
**Interview time**			
Written vs. Spoken	*F*_(1, 50)_ = 123.684, *p* < 0.001, *$\eta_{p}^{2}$*= 0.712 (power = 1.00)	Written: *M* = 1069.91, *SD* = 347.71 Spoken: *M* = 303.00, *SD* = 118.00	Written: 978.02–1161.80 Spoken: 271.82–334.19
**Word counts**			
Written vs. Spoken	*F*_(1, 53)_ = 3.026, *p* = 0.088, *$\eta_{p}^{2}$* = 0.052 (power = 0.40)	Written: *M* = 409.89, *SD* = 113.32 Spoken: *M* = 507.03, *SD* = 273.64	Written: 379.94–439.84 Spoken: 434.71–579.35

All *F*-tests for each of the seven dependent variables are reported in the second column of the table as a function of either writing or speaking during Phase 1. Participants who rewatched the video were not interviewed during this phase. All descriptive statistics (means and standard deviations) have been included along with the 95% confidence interval.

In addition to the number of details correctly reported during Phase 1, the accuracy of those details was assessed. Thus, input- and output-bound scores (Koriat and Goldsmith, 1994) were computed for each participant and then compared across conditions. Input-bound accuracy (IBA) is the proportion of central and peripheral details correctly reported. It was computed by dividing the number of correctly recalled central and peripheral details by the total number of predetermined central and peripheral details. Other details correctly reported are not considered in this metric. Output-bound accuracy (OBA) is the proportion of all details that are reported correctly. It was computed by dividing the number of correctly recalled details by the total amount of details recalled. This metric includes *all* correctly recalled details and any intrusions. It is important to note that with movie scenes, it is possible to report an infinite number of details (Koriat and Goldsmith, 1996), which might suggest that an IBA score is not appropriate to report. However, by relying on our norming study, we established that (one estimate of) the maximum number of central and peripheral details reported are 21 and 18 details, respectively. These are the details we included in our calculation of IBA.

**Figure 2:**
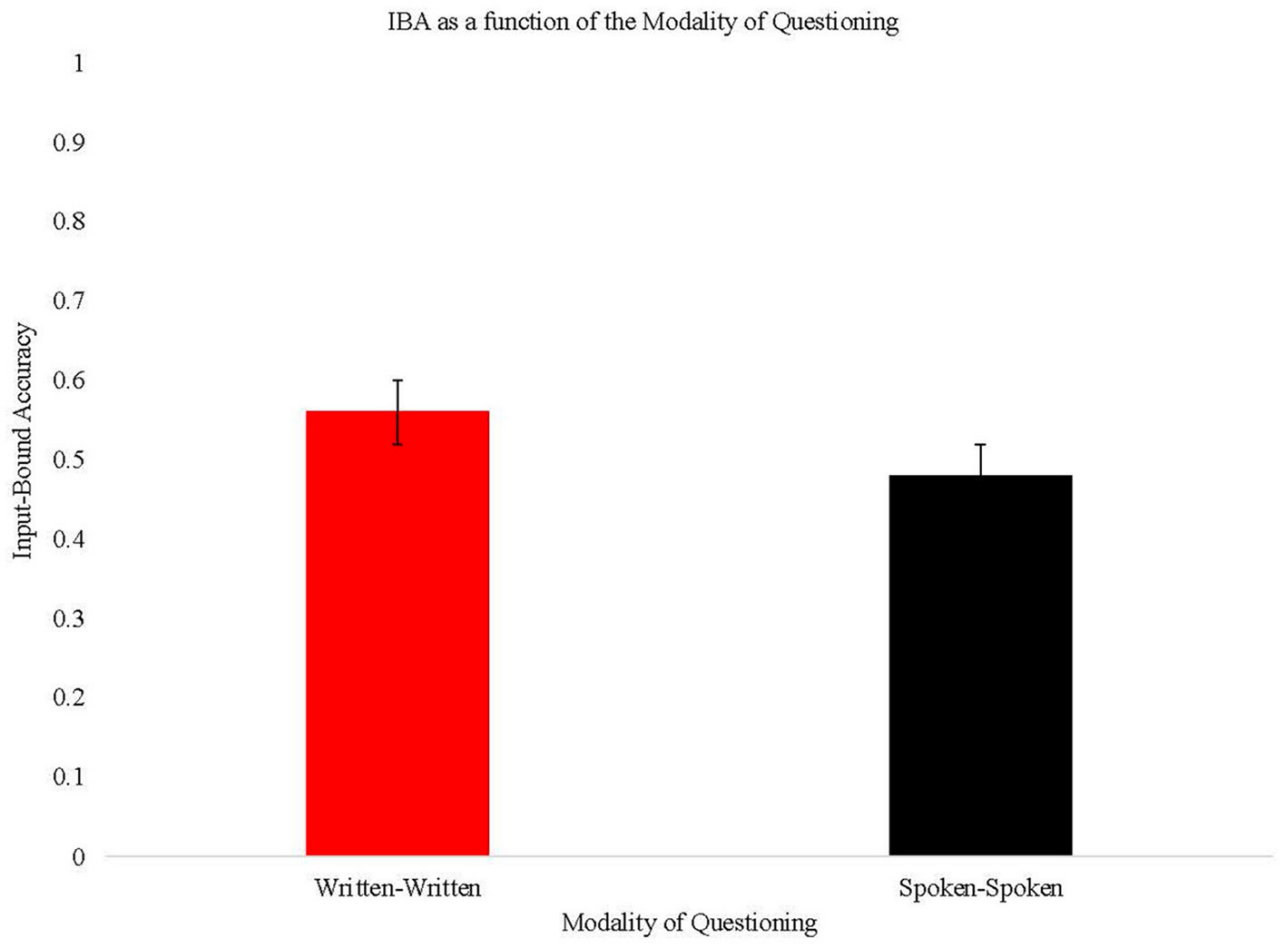
Experiment 1: Phase 1. IBA is portrayed as a function of the modality of questioning. Along the x-axis, participants in the Written-Written condition were those who were immediately questioned following the video and then wrote their answers during the interview. Participants in the Spoken-Spoken condition were those who were immediately questioned following the video and then wrote their answers during the interview. This figure illustrates the presence of the written superiority effect for Phase 1. Error bars represent standard errors.

Multiple one-way ANOVAs were conducted to compare IBA and OBA, completion times, and word counts for the Written-Written and Spoken-Spoken conditions. There was a significant difference in IBA and completion times, but not for OBA or word counts. More specifically, participants in the Written-Written condition (*M* = 0.56, *SD* = 0.12) had a greater IBA proportion than those in the Spoken-Spoken condition (*M* = 0.48, *SD* = 0.16). These findings suggest that, in general, writing immediately following encoding improves memory reports compared to speaking about what transpired. This effect is illustrated in Figure 2. Additionally, participants in the Written-Written condition (*M* = 1,069.91, *SD* = 347.71) took significantly more time to answer the questions than those in the Spoken-Spoken condition (*M* = 303.00, *SD* = 118.00). This finding is not surprising, given that writing is slower than speaking. However, combined with no difference in the number of words produced during Phase 1, these findings suggest that participants in the Written-Written condition are not performing better merely because they took more time to answer the questions. Instead, it suggests that participants are working equally hard in each condition, but participants in the Written-Written condition are working more effectively and accurately. All *F-tests* and relevant descriptive statistics are reported in Table 1.

#### Phase 2

Multiple two-way ANOVAs and subsequent pairwise comparisons with Bonferroni corrections were conducted on the Phase 2 data to understand better the effects that the modality of questioning and testing vs. restudying had on memory report changes over time. These ANOVAs were conducted to compare (1) IBA, (2) OBA, (3) the proportion of true pointed questions answered correctly, (4) the proportion of false questions endorsed, (5) the proportion of false questions rejected, (6) the time it took to complete the interview, and (7) word counts.

There was no significant main effect of the modality of questioning or testing vs. restudying, as well as no significant interaction for the dependent variables of IBA, OBA, or the proportion of true pointed questions answered correctly. All *F-tests* and relevant descriptive statistics are reported in Table 2. Though there was not a significant difference in OBA, it is important to note that participants were highly accurate in what they reported (see Table 2).

**Table 2:** Experiment 1: Phase 2 *F*-*tests* and relevant descriptive statistics.

**Dependent variable**	**Statistical test**	**Descriptive statistics**	***95% C.I*.**
**IBA**			
Modality of questioning	*F*_(1, 111)_ = 2.559, *p* = 0.113, *$\eta_{p}^{2}$*= 0.023 (power = 0.37)	Written: *M* = 0.47, *SD* = 0.13 Spoken: *M* = 0.43, *SD* = 0.14	Written: 0.43 −0.50 Spoken: 0.40 −0.46
Test vs. Restudy	*F*_(1, 111)_ = 1.435, *p* = 0.233, *$\eta_{p}^{2}$*= 0.013 (power = 0.23)	Test: *M* = 0.46, *SD* = 0.14 Restudy: *M* = 0.43, *SD* = 0.12	Test: 0.43 −0.50 Restudy: 0.40 −0.47
Interaction	*F*_(1, 111)_ = 3.047, *p* = 0.084, *$\eta_{p}^{2}$*= 0.027 (power = 0.43)		
**OBA**			
Modality of questioning	*F*_(1, 101)_ = 0.091, *p* = 0.763, *$\eta_{p}^{2}$*= 0.001 (power = 0.06)	Written: *M* = 0.89, *SD* = 0.06 Spoken: *M* = 0.89, *SD* = 0.06	Written: 0.88–0.90 Spoken: 0.88–0.90
Test vs. Restudy	*F*_(1, 101)_ = 0.270, *p* = 0.605, *$\eta_{p}^{2}$*= 0.003 (power = 0.09)	Test: *M* = 0.89, *SD* = 0.07 Restudy: *M* = 0.88, *SD* = 0.06	Test: 0.88–0.90 Restudy: 0.87–0.89
Interaction	*F*_(1, 101)_ = 2.329, *p* = 0.130, *$\eta_{p}^{2}$*= 0.023 (power = 0.34)		
**True pointed questions**			
Modality of questioning	*F*_(1, 111)_ = 0.042, *p* = 0.838, *$\eta_{p}^{2}$*= 0.000	Written: *M* = 0.83, *SD* = 0.11 Spoken: *M* = 0.83, *SD* = 0.14	Written: 0.80 −0.88 Spoken: 0.80 −0.86
Test vs. Restudy	*F*_(1, 111)_ = 2.488, *p* = 0.118, *$\eta_{p}^{2}$*= 0.022 (power = 0.36)	Test: *M* = 0.85, *SD* = 0.13 Restudy: *M* = 0.81, *SD* = 0.13	Test: 0.82–0.88 Restudy: 0.78–0.85
Interaction	*F*_(1, 111)_ = 0.048, *p* = 0.827, *$\eta_{p}^{2}$*= 0.000		
**False questions endorsed**			
Modality of questioning	*F*_(1, 111)_ = 7.118, *p* = 0.009, *$\eta_{p}^{2}$*= 0.060 (power = 0.77)	Written: *M* = 0.35, *SD* = 0.27 Spoken: *M* = 0.22, *SD* = 0.25	Written: 0.28–0.42 Spoken: 0.16–0.29
Test vs. Restudy	*F*_(1, 111)_ = 4.995, *p* = 0.027, *$\eta_{p}^{2}$*= 0.043 (power = 0.62)	Test: *M* = 0.34, *SD* = 0.26 Restudy: *M* = 0.23, *SD* = 0.27	Test: 0.27–0.41 Restudy: 0.17–0.30
Interaction	*F*_(1, 111)_ = 0.258, *p* = 0.613, *$\eta_{p}^{2}$*= 0.002 (power = 0.08)		
**False questions rejected**			
Modality of questioning	*F*_(1, 111)_ = 6.523, *p* = 0.012, *$\eta_{p}^{2}$*= 0.056 (power = 0.74)	Written: *M* = 0.27, *SD* = 0.25 Spoken: *M* = 0.16, *SD* = 0.20	Written: 0.21–0.33 Spoken: 0.10–0.22
Test vs. Restudy	*F*_(1, 111)_ = 5.709, *p* = 0.019, *$\eta_{p}^{2}$*= 0.049 (power = 0.67)	Test: *M* = 0.17, *SD* = 0.21 Restudy: *M* = 0.27, *SD* = 0.25	Test: 0.11–0.22 Restudy: 0.21–0.32
Interaction	*F*_(1, 111)_ = 0.821, *p* = 0.367, *$\eta_{p}^{2}$*= 0.007 (power = 0.15)		
**Interview Time**			
Modality of Questioning	*F*_(1, 111)_ = 411.1, *p* < 0.001, *$\eta_{p}^{2}$*= 0.787 (power = 1.00)	Written: *M* = 1,213.35, *SD* = 271.54 Spoken: *M* = 446.190, *SD* = 103.77	Written: 1,158.69–1,266.99 Spoken: 393.76–497.48
Test vs. Restudy	*F*_(1, 111)_ = 1.406, *p* = 0.238, *$\eta_{p}^{2}$*= 0.013 (power = 0.23)	Test: *M* = 793.29, *SD* = 430.84 Restudy: *M* = 831.89, *SD* = 440.48	Test: 753.11–860.48 Restudy: 799.33–904.01
Interaction	*F*_(1, 111)_ = 0.080, *p* = 0.777, *$\eta_{p}^{2}$*= 0.001 (power = 0.06)		
**Word counts**			
Modality of questioning	*F*_(1, 110)_ = 22.044, *p* < 0.001, *$\eta_{p}^{2}$*= 0.167 (power = 0.99)	Written: *M* = 408.81, *SD* = 114.40 Spoken: *M* = 572.74, *SD* = 234.62	Written: 360.09–457.77 Spoken: 523.72–621.39
Test vs. Restudy	*F*_(1, 110)_ = 0.010, *p* = 0.920, *$\eta_{p}^{2}$*= 0.000	Test: *M* = 488.98, *SD* = 189.63 Restudy: *M* = 492.50, *SD* = 213.71	Test: 439.72–538.25 Restudy: 444.09–540.91
Interaction	*F*_(1, 110)_ = 0.247, *p* = 0.621, *$\eta_{p}^{2}$*= 0.002 (power = 0.08)		

All *F*-tests for each of the seven dependent variables are reported in the second column of the table as a function of the main effects for both the modality of questioning and testing vs. restudying, as well as the interactions. All descriptive statistics (means and standard deviations) have been included along with the 95% confidence interval.

For the proportion of false questions endorsed, there was a significant main effect of the modality of questioning. More specifically, participants who wrote (*M* = 0.35, *SD* = 0.27) endorsed false questions to a greater extent than participants who spoke (*M* = 0.22, *SD* = 0.25). Here, endorsing a false question is an incorrect response (participants should indicate that the question asked about something that did not happen). Therefore, participants who wrote did worse than participants who spoke when answering the false questions. There was also a significant main effect of testing vs. restudying, such that participants who were interviewed immediately (Test) (*M* = 0.34, *SD* = 0.26) endorsed false questions to a greater extent than participants who had the opportunity to rewatch the video (Restudy) (*M* = 0.23, *SD* = 0.27). There was not a significant interaction between the modality of questioning and testing vs. restudying. All *F-tests* and relevant descriptive statistics are reported in Table 2.

There was also a significant main effect of the proportion of false questions correctly rejected. Participants who wrote (*M* = 0.27*, SD* = 0.25) correctly rejected the false questions as asking about a false detail more than participants who spoke (*M* = 0.16*, SD* = 0.20). Additionally, there was a significant main effect of testing vs. restudying such that participants who had the opportunity to rewatch the video (*M* = 0.27, *SD* = 0.25) correctly rejected the false questions more than participants who were immediately questioned (*M* = 0.17, *SD* = 0.21) about the video. This suggests that, in general, restudying (vs. immediate testing) led to better performance regarding handling the false questions, consistent with research supporting that restudying promotes verbatim recall. There was no significant interaction between the modality of questioning and testing vs. restudying. All *F-tests* and relevant descriptive statistics are reported in Table 2.

Unsurprisingly, there was a significant main effect of the interview completion times such that participants who wrote (*M* = 1,213.35, *SD* = 271.54) took significantly more time to complete the interview compared to participants who spoke (*M* = 446.190, *SD* = 103.77), replicating the results from Phase 1 (*p*'s < 0.05). There was not a significant main effect of testing vs. restudying or an interaction. Of greater interest are the significant differences in the number of words produced during the interviews. Participants who wrote (*M* = 408.81, *SD* = 114.40) produced fewer words than those who spoke (*M* = 572.74, *SD* = 234.62). There was no significant main effect of testing vs. restudying or a significant interaction. All *F-tests* and relevant descriptive statistics are reported in Table 2.

#### Phase 3

The Phase 3 data were checked for outliers, normality tests were conducted, and the relevant data were transformed, when necessary, via log transformations. The time to answer the questions was not recorded for Phase 3 because the questioning occurred via email. Multiple two-way ANOVAs and subsequent pairwise comparisons with Bonferroni corrections were conducted on the Phase 3 data to understand better the effects that the modality of questioning and testing vs. restudying had on memory report changes over time. These ANOVAs were conducted to compare (1) IBA, (2) OBA, (3) the proportion of true pointed questions answered correctly, (4) the proportion of false questions endorsed, (5) the proportion of false questions rejected, (6) open-ended question word counts, and (7) pointed-question word counts. These analyses all produced non-significant findings (*p*'s > 0.05) except for a significant main effect of testing vs. restudying for the proportion of false questions correctly rejected. Those who had the opportunity to restudy (*M* = 0.26, *SD* = 0.26) correctly rejected the false questions as asking about a false detail more than participants who were immediately questioned (*M* = 0.16, *SD* = 0.21). These findings suggest that the written superiority effect only extended to 1 week after encoding. All *F-tests* and relevant descriptive statistics are reported in Table 3.

**Table 3:** Experiment 1: Phase 3 *F*-*tests* and relevant descriptive statistics.

**Dependent variable**	**Statistical test**	**Descriptive statistics**	***95% C.I*.**
**IBA**			
Modality of questioning	*F*_(1, 87)_ = 2.921, *p* = 0.091, *$\eta_{p}^{2}$*= 0.032 (power = 0.40)	Written: *M* = 0.42, *SD* = 0.15 Spoken: *M* = 0.37, *SD* = 0.14	Written: 0.38–0.46 Spoken: 0.32–0.41
Test vs. Restudy	*F*_(1, 87)_ = 0.175, *p* = 0.677, *$\eta_{p}^{2}$*= 0.002 (power = 0.07)	Test: *M* = 0.40, *SD* = 0.15 Restudy: *M* = 0.39, *SD* = 0.14	Test: 0.36–0.44 Restudy: 0.34–0.43
Interaction	*F*_(1, 87)_ = 1.518, *p* = 0.221, *$\eta_{p}^{2}$*= 0.017 (power = 0.24)		
**OBA**			
Modality of Questioning	*F*_(1, 80)_ = 0.436, *p* = 0.511, *$\eta_{p}^{2}$*= 0.005 (power = 0.1)	Written: *M* = 0.89, *SD* = 0.06 Spoken: *M* = 0.88, *SD* = 0.07	Written: 0.87–0.91 Spoken: 0.86–0.90
Test vs. Restudy	*F*_(1, 80)_ = 0.057, *p* = 0.811, *$\eta_{p}^{2}$*= 0.001 (power = 0.06)	Test: *M* = 0.89, *SD* = 0.06 Restudy: *M* = 0.88, *SD* = 0.06	Test: 0.87–0.91 Restudy: 0.86–0.90
Interaction	*F*_(1, 80)_ = 0.945, *p* = 0.334, *$\eta_{p}^{2}$*= 0.012 (power = 0.17)		
**True Pointed Questions**			
Modality of Questioning	*F*_(1, 84)_ = 0.354, *p* = 0.554, *$\eta_{p}^{2}$*= 0.004 (power = 0.09)	Written: *M* = 0.83, *SD* = 0.12 Spoken: *M* = 0.85, *SD* = 0.13	Written: 0.79–0.87 Spoken: 0.81–0.88
Test vs. Restudy	*F*_(1, 84)_ = 0.417, *p* = 0.520, *$\eta_{p}^{2}$*= 0.005 (power = 0.10)	Test: *M* = 0.85, *SD* = 0.14 Restudy: *M* = 0.83, *SD* = 0.12	Test: 0.81–0.88 Restudy: 0.79–0.87
Interaction	*F*_(1, 84)_ = 0.751, *p* = 0.389, *$\eta_{p}^{2}$*= 0.009 (power = 0.14)		
**False questions endorsed**			
Modality of questioning	*F*_(1, 87)_ = 0.158, *p* = 0.692, *$\eta_{p}^{2}$*= 0.002 (power = 0.07)	Written: *M* = 0.42, *SD* = 0.30 Spoken: *M* = 0.39, *SD* = 0.29	Written: 0.33–0.50 Spoken: 0.31–0.48
Test vs. Restudy	*F*_(1, 87)_ = 1.716, *p* = 0.194, *$\eta_{p}^{2}$*= 0.019 (power = 0.26)	Test: *M* = 0.44, *SD* = 0.30 Restudy: *M* = 0.37, *SD* = 0.29	Test: 0.36–0.53 Restudy: 0.28–0.45
Interaction	*F*_(1, 87)_ = 2.543, *p* = 0.114, *$\eta_{p}^{2}$*= 0.028 (power = 0.36)		
**False questions rejected**			
Modality of questioning	*F*_(1, 87)_ = 0.530, *p* = 0.469, *$\eta_{p}^{2}$*= 0.006 (power = 0.11)	Written: *M* = 0.23, *SD* = 0.25 Spoken: *M* = 0.19, *SD* = 0.23	Written: 0.16–0.30 Spoken: 0.12–0.26
Test vs. Restudy	*F*_(1, 87)_ = 3.868, *p* = 0.052, *$\eta_{p}^{2}$*= 0.04 (power = 0.49)	Test: *M* = 0.16, *SD* = 0.21 Restudy: *M* = 0.26, *SD* = 0.26	Test: 0.09–0.33 Restudy: 0.19–0.33
Interaction	*F*_(1, 87)_ = 0.261, *p* = 0.611, *$\eta_{p}^{2}$*= 0.003 (power = 0.08)		
**Open-ended word counts**			
Modality of questioning	*F*_(1, 85)_ = 3.513, *p* = 0.064, *$\eta_{p}^{2}$*= 0.040 (power = 0.48)	Written: *M* = 328.09, *SD* = 146.69 Spoken: *M* = 274.16, *SD* = 120.17	Written: 287.87–368.38 Spoken: 233.46–314.86
Test vs. Restudy	*F*_(1, 85)_ = 0.002, *p* = 0.965, *$\eta_{p}^{2}$*= 0.000	Test: *M* = 300.50, *SD* = 140.09 Restudy: *M* = 302.33, *SD* = 133.88	Test: 259.80–341.20 Restudy: 261.53–342.04
Interaction	*F*_(1, 85)_ = 0.022, *p* = 0.881, *$\eta_{p}^{2}$*= 0.000		
**Pointed question word counts**			
Modality of questioning	*F*_(1, 84)_ = 1.487, *p* = 0.226, *$\eta_{p}^{2}$*= 0.017 (power = 0.23)	Written: *M* = 72.73, *SD* = 24.31 Spoken: *M* = 79.20, *SD* = 26.55	Written: 65.10–80.37 Spoken: 71.71–87.00
Test vs. Restudy	*F*_(1, 84)_ = 0.023, *p* = 0.880, *$\eta_{p}^{2}$*= 0.000	Test: *M* = 75.47, *SD* = 25.49 Restudy: *M* = 76.44, *SD* = 25.82	Test: 67.9–83.36 Restudy: 68.90–84.00
Interaction	*F*_(1, 84)_ = 1.833, *p* = 0.179, *$\eta_{p}^{2}$*= 0.021 (power = 0.27)		

All *F*-tests for each of the seven dependent variables are reported in the second column of the table as a function of the main effects for both the modality of questioning and testing vs. restudying, as well as the interactions. All descriptive statistics (means and standard deviations) have been included along with the 95% confidence interval.

#### Performance of participants who completed all sessions

It is possible that the number of participants that self-selected out at each phase could have impacted the previous findings. Therefore, two 2 × 2 × 2 mixed ANOVAs and subsequent pairwise comparisons with Bonferroni corrections were conducted for the IBAs and OBAs as a function of the modality of questioning and testing vs. restudying to assess potential differences across phases that may have been obscured by only examining participants who completed at least Phase 1 and Phase 2. Thus, the following analyses involve only participants who completed every phase (*N* = 91). There was a significant difference in IBA across phases such that the IBAs were significantly lower from Phase 2 (*M* = 0.44, *SD* = 0.14) to Phase 3 (*M* = 0.39, *SD* = 0.15). There were no other significant within-subject main effects or interactions, or any significant between-subject main effects or interactions (*p*'s > 0.05). When examining OBAs, there were no significant between- or within-subject main effects or interactions. All *F-tests* and relevant descriptive statistics are reported in Table 4.

**Table 4:** Experiment 1: Performance of participants who completed all sessions.

**Dependent variable**	**Statistical test**	**Descriptive statistics**	***95% C.I*.**
**IBA**			
**Between-subject effects**			
Modality of questioning	*F*_(1, 87)_ = 3.669, *p* = 0.059, *$\eta_{p}^{2}$*= 0.04 (power = 0.49)		
Test vs. Restudy	*F*_(1, 87)_ = 0.921, *p* = 0.340, *$\eta_{p}^{2}$*= 0.010 (power = 0.16)		
Interaction	*F*_(1, 87)_ = 2.063, *p* = 0.155, *$\eta_{p}^{2}$*= 0.023 (power = 0.30)		
**Within-subject effects**			
Timing	*F*_(1, 87)_ = 11.120, *p* = 0.001, *$\eta_{p}^{2}$*= 0.113 (power = 0.92)	Phase 2: *M* = 0.44, *SD* = 0.14 Phase 3: *M* = 0.39, *SD* = 0.15	Phase 2: 0.41–0.47 Phase 3: 0.36–0.42
Timing^*^modality of questioning	*F*_(1, 87)_ = 0.040, *p* = 0.841, *$\eta_{p}^{2}$*= 0.000	Phase 2^*^Written: *M* = 0.46, *SD* = 0.13 Phase 2^*^Spoken: *M* = 0.42, *SD* = 0.14 Phase 3^*^Written: *M* = 0.42, *SD* = 0.15 Phase 3^*^Spoken: *M* = 0.37, *SD* = 0.14	Phase 2^*^Written: 0.43–0.50 Phase 2^*^Spoken: 0.38–0.46 Phase 3^*^Written: 0.38–0.46 Phase 3^*^Spoken: 0.32–0.41
Timing^*^test vs. restudy	*F*_(1, 87)_ = 0.660, *p* = 0.419, *$\eta_{p}^{2}$*= 0.008 (power = 0.14)	Phase 2^*^Test: *M* = 0.46, *SD* = 0.14 Phase 2^*^Restudy: *M* = 0.42, *SD* = 0.13 Phase 3^*^Test: *M* = 0.40, *SD* = 0.15 Phase 3^*^Restudy: *M* = 0.37, *SD* = 0.14	Phase 2^*^Test:0.42–0.50 Phase 2^*^Restudy: 0.38–0.46 Phase 3^*^Test: 0.36–0.44 Phase 3^*^Restudy: 0.34–0.43
Interaction	*F*_(1, 87)_ = 0.002, *p* = 0.962, *$\eta_{p}^{2}$*= 0.000		
**OBA**			
**Between-subject effects**			
Modality of questioning	*F*_(1, 73)_ = 0.005, *p* = 0.946, *$\eta_{p}^{2}$*= 0.000		
Test vs. Restudy	*F*_(1, 73)_ = 0.066, *p* = 0.798, *$\eta_{p}^{2}$*= 0.001 (power = 0.06)		
Interaction	*F*_(1, 73)_ = 2.730, *p* = 0.103, *$\eta_{p}^{2}$*= 0.036 (power = 0.39)		
**Within-subject effects**			
Timing	*F*_(1, 73)_ = 0.008, *p* = 0.928, *$\eta_{p}^{2}$*= 0.000	Phase 2: *M* = 0.89, *SD* = 0.07 Phase 3: *M* = 0.89, *SD* = 0.06	Phase 2: 0.87–0.90 Phase 3: 0.87–0.90
Timing^*^modality of questioning	*F*_(1, 73)_ = 0.866, *p* = 0.355, *$\eta_{p}^{2}$*= 0.012 (power = 0.16)	Phase 2^*^Written: *M* = 0.88, *SD* = 0.07 Phase 2^*^Spoken: *M* = 0.89, *SD* = 0.07 Phase 3^*^Written: *M* = 0.89, *SD* = 0.05 Phase 3^*^Spoken: *M* = 0.88, *SD* = 0.06	Phase 2^*^Written: 0.86–0.90 Phase 2^*^Spoken: 0.87–0.91 Phase 3^*^Written: 0.87–0.91 Phase 3^*^Spoken: 0.86–0.90
Timing^*^test vs. restudy	*F*_(1, 73)_ = 0.285, *p* = 0.595, *$\eta_{p}^{2}$*= 0.004 (power = 0.09)	Phase 2^*^Test: *M* = 0.89, *SD* = 0.07 Phase 2^*^Restudy: *M* = 0.88, *SD* = 0.06 Phase 3^*^Test: *M* = 0.88, *SD* = 0.06 Phase 3^*^Restudy: *M* = 0.89, *SD* = 0.06	Phase 2^*^Test:0.87–0.91 Phase 2^*^Restudy: 0.86–0.90 Phase 3^*^Test: 0.87–0.91 Phase 3^*^Restudy: 0.87–0.90
Interaction	*F*_(1, 73)_ = 2.865, *p* = 0.095, *$\eta_{p}^{2}$*= 0.038 (power = 0.41)		

All *F*-tests for each of the two dependent variables are reported in the second column of the table as a function of the main effects for the modality of questioning, testing vs. restudying, and timing (Phase 2 and Phase 3), as well as the interactions. All descriptive statistics (means and standard deviations) have been included along with the 95% confidence interval.

#### Rate of information loss

**Figure 3:**
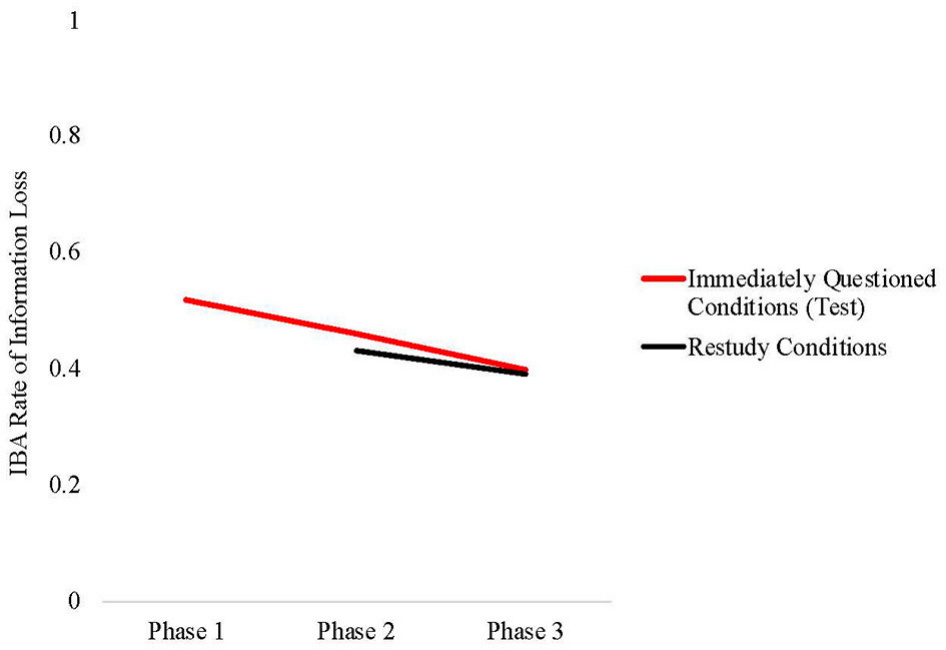
Experiment 1: Rate of information loss for IBA as a function of phase and test vs. restudy. The rate of information loss for IBA is presented along the y-axis as a function of each phase and whether a participant was immediately questioned or rewatched the video as a function of time (Phase). Over time, IBA decreases. Phase 2 occurs 1 week after Phase 1, and Phase 3 occurs 1 month after Phase 1.

Multiple two-way ANOVAs and subsequent pairwise comparisons with Bonferroni corrections were conducted on the rate of information loss from Phase 1 to Phase 2 to Phase 3 for the immediately questioned groups and the rate of information loss from Phase 2 to Phase 3 for the restudy groups. There was a significant difference in the rate of information loss for IBA for participants in the immediately questioned groups [*F*_(5, 153)_ = 6.429, *p* < 0.001, $\eta_{p}^{2}$ = 0.174] (power = 1.00), such that participants had a significantly lower IBA in Phase 3 (*M* = 0.40, *SD* = 0.15) compared to Phase 1 (*M* = 0.52, *SD* = 0.15); [*F*_(2, 153)_ = 9.199, *p* < 0.001, $\eta_{p}^{2}$ = 0.107] (power = 1.00) (Figure 3). There was also a significant difference in IBA for those who wrote (*M* = 0.51, *SD* = 0.14) vs. spoke (*M* = 0.42, *SD* = 0.15); [*F*_(1, 153)_ = 14.159, *p* < 0.001, $\eta_{p}^{2}$ = 0.085] (power = 1.00). There was not a significant interaction between Phase and the immediately questioned groups (Written or Spoken) [*F*_(2, 153)_ = 0.009, *p* = 0.991, $\eta_{p}^{2}$ = 0.000]. There was not a significant difference in the rate of information loss for OBA for participants immediately questioned across the three phases: *F*_(5, 139)_ = 1.254, *p* = 0.288, $\eta_{p}^{2}$= 0.043. For the restudy groups, there was not a significant difference in the rate of information loss for IBA [*F*_(3, 100)_ = 1.126, *p* = 0.342, $\eta_{p}^{2}$ = 0.033]. Additionally, there was no significant difference in the rate of information loss for OBA: *F*_(3, 94)_ = 0.659, *p* = 0.580, $\eta_{p}^{2}$ = 0.021.

### Discussion

Results from the present experiment are consistent with research supporting a written superiority effect in Phase 1 and 2 (Kraus et al., 2017; Sauerland et al., 2014). Participants who write provide more correct information compared to their counterparts, although the amount of information they produce is similar. Sauerland and Sporer (2011) posited that speaking may be more productive but not necessarily as efficient; the present study provides support for this idea based on the longer completion times for those participants who wrote but equivalent word counts compared to those who spoke.

In general, restudying led to better performance regarding the management of false questions, consistent with research supporting that restudying promotes verbatim recall (Reyna and Brainerd, 1995). However, the better performance operated differently depending on whether participants wrote or spoke following restudying. There may be a differential criterion shift such that participants' inclination to provide an answer increased in the written conditions, possibly due to not having to directly provide answers to the research assistant, whereas those who spoke may have been reticent to provide an incorrect answer directly to the research assistant. These findings provide another indication that the benefit due to writing can override an anticipated testing effect.

## Experiment 2

Experiment 2 was conducted to replicate and extend the findings of Experiment 1. The COVID-19 pandemic created a natural experiment examining the impact of removing some of the social factors that may impact reporting performance. Experiment 2 was identical to Experiment 1 with the exception that it occurred virtually using the platform Zoom rather than in-person. Prior research suggests that allowing individuals to be questioned remotely can lead to an increase in the accuracy of overall memory reports and a reduction in error reporting (Nash et al., 2014; Taylor and Dando, 2018). Therefore, it is possible that the written superiority effect seen in Experiment 1 could be enhanced in Experiment 2 due to removing the presence of the interviewer.

In addition to the cognitive factors that may impact memory retrieval, there are social factors that impact performance, like the presence of an interviewer. Bergmann et al. (2004) had patients complete a written questionnaire and personal interview related to their medical history. They found that when the interviewer was absent, the reporting of serious diseases was less likely. In a review, Rosenthal (2002) suggested that the presence of an interviewer may unintentionally introduce cues to the witnesses who spoke to report more central rather than peripheral details, though the interviewer was not instructed to do so. This might occur because the interviewer's presence may increase an individual's overall motivation to perform, thereby focusing on more relevant information. Sauerland et al. (2014) found that conditions in which the interviewer was absent while writing led to better recall performance. We do not expect the move to virtual testing to influence the testing benefit. Lastly, we still expect participants who restudy the information, instead of being immediately questioned, to be less likely to endorse the false questions.

In Experiment 2, both the researcher and participant kept their cameras turned off. It is important to note that because the experimental sessions occurred virtually, the participants typed their responses instead of writing on lined sheets of paper. However, previous research has shown that typing and writing lead to similar performance levels on essay exams. Though typing is faster, the quality of the essays is not significantly different (Mogey et al., 2010).

### Method

#### Participants

A statistical power analysis was conducted using GPower to determine the sample size, which was based on Experiment 1's effect size estimate of 0.35 (power = 0.95), which determined an ideal sample size of 109 participants. However, a total of 84 introductory psychology students (32 males, 52 females; *M*_*Age*_ = 20.04 years, *SD*_Age_ = 4.54) from the University of Oklahoma participated in this study in exchange for partial course credit, therefore resulting in an overall *post hoc* power estimate of 0.89. All students were recruited via a university recruitment portal (SONA study flier). The flier informed potential participants that they would watch a video and be asked various questions about the video at three different time points. Participants received a maximum of 2.5 research credits for their psychology course. They received credit following the completion of two virtual sessions and one email response. To participate, students must have been at least 18 years of age and be able to provide consent. In addition, participants must have considered themselves proficient in English.

As in Experiment 1, participants were randomly assigned to: Restudy-Written (*n* = 19), Restudy-Spoken (*n* = 20), Written-Written (*n* = 25), and Spoken-Spoken (*n* = 20). All participants' data were kept anonymous and separate from all possible identifying information. No significant risks were encountered by the participants, and they were treated in accordance with APA ethical standards. This study was approved by the University of Oklahoma IRB.

### Materials

The materials used in this experiment were identical to those used in Experiment 1.

#### Design and procedure

The design of Experiment 2 is the same as Experiment 1. A 2 (Modality of Questioning: Written vs. Spoken) × 2 (Test vs. Restudy) × 3 (Timing: Immediate, 1-week delay, and 1-month delay, hereafter denoted Phase 1, Phase 2, and Phase 3) incomplete factorial design was used. The dependent variables are the same as in Experiment 1.

Experiment 2′s procedure is identical to Experiment 1, apart from it occurring virtually. Before beginning the sessions, participants were instructed to turn their computer cameras off. The researchers also kept their computer camera off. The researchers shared their screen to show the participant the video. Also, instead of recording participants' spoken answers with an audio recorder as in Experiment 1, the virtual meeting was recorded and uploaded to MyMedia (OU MyMedia, 2020) for transcription, after which a researcher edited and fixed any transcription errors. Only participants in the Spoken conditions (Restudy-Spoken and Spoken-Spoken) had their answers recorded and uploaded to MyMedia. Participants were only recorded when they answered the questions; the parts of the session that occurred before the questions were asked were not recorded. Participants in the written conditions (Restudy-Written and Written-Written) typed their responses in a Word document instead of writing on lined sheets of paper. These participants emailed their responses immediately to the researcher when the session ended. The responses were de-identified and saved.

### Results

Eighty four participants completed Phase 1. Of those, 75 (89.3% return rate) participants completed Phase 2, and 44 (52.4% response rate) participants completed Phase 3. Only participants who completed Phases 1 and 2 (*n* = 75) were included in the subsequent analyses. An additional participant was removed (failed to return their responses by the due date) for the Phase 3 analyses, so 43 (out of 44) participants' email responses were used for the Phase 3 analyses. All data were checked for outliers and transformed when necessary.

#### Phase 1

The total number of central, peripheral, and correct other details reported during Phase 1 were aggregated to reflect the overall correct number of details reported. A one-way ANOVA compared the total correct number of details reported in the Spoken-Spoken and Written-Written conditions. Participants in the Written-Written condition (*M* = 31.84, *SD* = 8.83) did not differ significantly in the number of correct details reported compared to the Spoken-Spoken condition (*M* = 26.70, *SD* = 10.39). The corresponding *F-test* and relevant descriptive statistics are reported in Table 5.

**Table 5:** Experiment 2: Phase 1 *F*-tests and relevant descriptive statistics.

**Dependent variable**	**Statistical test**	**Descriptive statistics**	***95% C.I*.**
**Total correct number of details reported**			
Written vs. Spoken	*F*_(1, 37)_ = 2.761, *p* = 0.105, *$\eta_{p}^{2}$*= 0.069 (power = 0.38)	Written: *M* = 31.84, *SD* = 8.83 Spoken: *M* = 26.70, *SD* = 10.39	Written: 29.07–34.61 Spoken: 23.44–29.96
**IBA**			
Written vs. Spoken	*F*(1, 39) = 1.995, *p* = 0.166, *$\eta_{p}^{2}$* = 0.049) (power = 0.29)	Written: *M* = 0.46, *SD* = 0.13 Spoken: *M* = 0.39, *SD* = 0.18	Written: 0.42–0.50 Spoken: 0.33–0.45
**OBA**			
Written vs. Spoken	*F*_(1, 37)_ = 0.043, *p* = 0.937, *$\eta_{p}^{2}$* = 0.000	Written: *M* = 0.90, *SD* = 0.06 Spoken: *M* = 0.88, *SD* = 0.09	Written: 0.88–0.92 Spoken: 0.85–0.91
**Interview time**			
Written vs. Spoken	*F*_(1, 40)_ = 41.828, *p* < 0.001, *$\eta_{p}^{2}$*= 0.511 (power = 0.99)	Written: *M* = 917.95, *SD* = 370.26 Spoken: *M* = 354.80, *SD* = 125.29	Written: 801.75–1034.15 Spoken: 315.78–394.12
**Word counts**			
Written vs. Spoken	*F*_(1, 39)_ = 0.547, *p* = 0.464, *$\eta_{p}^{2}$* = 0.014) (power = 0.12)	Written: *M* = 424.59, *SD* = 166.51 Spoken: *M* = 470.55, *SD* = 223.41	Written: 372.33–476.85 Spoken: 400.43–540.67

All *F*-tests for each of the five dependent variables are reported in the second column of the table as a function of either writing or speaking during Phase 1. Participants who rewatched the video were not interviewed during this phase. All descriptive statistics (means and standard deviations) have been included along with the 95% confidence interval.

Multiple one-way ANOVAs were conducted to compare the effect of the modality of questioning on IBA, OBA, completion times, and word count for the Written-Written and Spoken-Spoken conditions. Consistent with Experiment 1, participants in the Written-Written condition (*M* = 917.95, *SD* = 370.26) took significantly more time to answer the questions compared to those in the Spoken-Spoken condition (*M* = 354.80, *SD* = 125.29). However, there was no significant difference in IBA, OBA or word counts. Taken together, these findings suggest that, at least virtually, the modality of questioning did not significantly influence Phase 1 memory reports. All *F-tests* and relevant descriptive statistics are reported in Table 5.

#### Phase 2

Multiple two-way ANOVAs and subsequent pairwise comparisons with Bonferroni corrections were conducted on the Phase 2 data to understand better the effects that the modality of questioning and testing vs. restudying had on memory report changes over time. These ANOVAs were conducted to compare (1) IBA, (2) OBA, (3) the proportion of true pointed questions answered correctly, (4) the proportion of false questions endorsed, (5) the proportion of false questions rejected, (6) the time it took to complete the interview, and (7) word counts.

There was a significant main effect of the modality of questioning for IBA such that participants who wrote (*M* = 0.44, *SD* = 0.13) had a greater IBA than participants who spoke (*M* = 0.35, *SD* = 0.16). These findings are consistent with writing improving memory reports compared to speaking, at a 1-week delay. There was no significant main effect of testing vs. restudying or a significant interaction. All *F-tests* and relevant descriptive statistics are reported in Table 6.

**Table 6:** Experiment 2: Phase 2 *F*-tests and relevant descriptive statistics.

**Dependent variable**	**Statistical test**	**Descriptive statistics**	***95% C.I*.**
**IBA**			
Modality of questioning	*F*_(1, 71)_ = 7.840, *p* = 0.007, *$\eta_{p}^{2}$*= 0.099 (power = 0.81)	Written: *M* = 0.44, *SD* = 0.13 Spoken: *M* = 0.35, *SD* = 0.16	Written: 0.39–0.49 Spoken: 0.30–0.39
Test vs. Restudy	*F*_(1, 71)_ = 1.385, *p* = 0.243, *$\eta_{p}^{2}$*= 0.019 (power = 0.22)	Test: *M* = 0.37, *SD* = 0.16 Restudy: *M* = 0.41, *SD* = 0.14	Test: 0.33–0.42 Restudy: 0.37–0.46
Interaction	*F*_(1, 71)_ = 0.028, *p* = 0.868, *$\eta_{p}^{2}$*= 0.000		
**OBA**			
Modality of questioning	*F*_(1, 65)_ = 0.242, *p* = 0.624, *$\eta_{p}^{2}$*= 0.004 (power = 0.08)	Written: *M* = 0.87, *SD* = 0.06 Spoken: *M* = 0.88, *SD* = 0.06	Written: 0.86–0.89 Spoken: 0.86–0.90
Test vs. Restudy	*F*_(1, 65)_ = 0.005, *p* = 0.944, *$\eta_{p}^{2}$*= 0.000	Test: *M* = 0.88, *SD* = 0.06 Restudy: *M* = 0.88, *SD* = 0.05	Test: 0.86–0.90 Restudy: 0.86–0.90
Interaction	*F*_(1, 65)_ = 2.639, *p* = 0.109, *$\eta_{p}^{2}$*= 0.039 (power = 0.38)		
**True pointed questions**			
Modality of questioning	*F*_(1, 71)_ = 1.997, *p* = 0.162, *$\eta_{p}^{2}$*= 0.027 (power = 0.30)	Written: *M* = 0.80, *SD* = 0.11 Spoken: *M* = 0.76, *SD* = 0.12	Written: 0.76–0.84 Spoken: 0.73–0.80
Test vs. Restudy	*F*_(1, 71)_ = 0.784, *p* = 0.379, *$\eta_{p}^{2}$*= 0.011 (power = 0.15)	Test: *M* = 0.77, *SD* = 0.13 Restudy: *M* = 0.79, *SD* = 0.10	Test: 0.73–0.81 Restudy: 0.76–0.83
Interaction	*F*_(1, 71)_ = 1.522, *p* = 0.221, *$\eta_{p}^{2}$*= 0.021 (power = 0.24)		
**False questions endorsed**			
Modality of questioning	*F*_(1, 71)_ = 2.175, *p* = 0.145, *$\eta_{p}^{2}$*= 0.030 (power = 0.32)	Written: *M* = 0.33, *SD* = 0.28 Spoken: *M* = 0.24, *SD* = 0.23	Written: 0.24–0.41 Spoken: 0.16–0.32
Test vs. Restudy	*F*_(1, 71)_ = 2.481, *p* = 0.120, *$\eta_{p}^{2}$*= 0.034 (power = 0.36)	Test: *M* = 0.33, *SD* = 0.25 Restudy: *M* = 0.23, *SD* = 0.26	Test: 0.25–0.41 Restudy: 0.15–0.32
Interaction	*F*_(1, 71)_ = 0.238, *p* = 0.627, *$\eta_{p}^{2}$*= 0.003 (power = 0.08)		
**False questions rejected**			
Modality of questioning	*F*_(1, 71)_ = 0.031, *p* = 0.861, *$\eta_{p}^{2}$*= 0.000	Written: *M* = 0.31, *SD* = 0.28 Spoken: *M* = 0.30, *SD* = 0.29	Written: 0.22–0.40 Spoken: 0.21–0.39
Test vs. Restudy	*F*_(1, 71)_ = 3.188, *p* = 0.078, *$\eta_{p}^{2}$*= 0.043 (power = 0.44)	Test: *M* = 0.25, *SD* = 0.24 Restudy: *M* = 0.36, *SD* = 0.31	Test: 0.16–0.34 Restudy: 0.27–0.46
Interaction	*F*_(1, 71)_ = 0.373, *p* = 0.544, *$\eta_{p}^{2}$*= 0.005 (power = 0.09)		
**Interview time**			
Modality of questioning	*F*_(1, 69)_ = 70.650, *p* < 0.001, *$\eta_{p}^{2}$*= 0.506 (power = 1.00)	Written: *M* = 1,082.18, *SD* = 344.58 Spoken: *M* = 553.62, *SD* = 182.16	Written: 993.35–1,177.56 Spoken: 468.97–640.73
Test vs. Restudy	*F*_(1, 69)_ = 2.703, *p* = 0.105, *$\eta_{p}^{2}$*= 0.038 (power = 0.39)	Test: *M* = 754.50, *SD* = 393.95 Restudy: *M* = 848.97, *SD* = 357.65	Test: 681.17–855.36 Restudy: 781.08–963.00
Interaction	*F*_(1, 69)_ = 0.015, *p* = 0.903, *$\eta_{p}^{2}$*= 0.000		
**Word counts**			
Modality of questioning	*F*_(1, 68)_ = 6.531, *p* = 0.013, *$\eta_{p}^{2}$*= 0.088 (power = 0.74)	Written: *M* = 461.19, *SD* = 204.43 Spoken: *M* = 596.36, *SD* = 255.98	Written: 383.23–537.11 Spoken: 522.58–676.46
Test vs. Restudy	*F*_(1, 68)_ = 0.492, *p* = 0.485, *$\eta_{p}^{2}$*= 0.007 (power = 0.11)	Test: *M* = 510.71, *SD* = 255.34 Restudy: *M* = 548.97, *SD* = 223.36	Test: 435.94–585.48 Restudy: 469.92–628.02
Interaction	*F*_(1, 68)_ = 1.907, *p* = 0.172, *$\eta_{p}^{2}$*= 0.027 (power = 0.29)		

All *F*-tests for each of the seven dependent variables are reported in the second column of the table as a function of the main effects for both the modality of questioning and testing vs. restudying, as well as the interactions. All descriptive statistics (means and standard deviations) have been included along with the 95% confidence interval.

There was no significant main effect of the modality of questioning or test vs. restudy or significant interaction for OBA and the proportion of true pointed questions answered correctly, which is consistent with Experiment 1. But contrary to Experiment 1, there was no significant main effect of the modality of questioning or test vs. restudy or significant interaction for the proportion of false questions endorsed and the proportion of false questions correctly rejected. Therefore, there was no differential effect of the modality of questioning or testing vs. restudying when answering the pointed questions. All *F-tests* and relevant descriptive statistics are reported in Table 6.

Unsurprisingly, there was a significant main effect of the interview completion times such that participants who wrote (*M* = 1,082.18, *SD* = 344.58) took significantly more time to complete the interview compared to participants who spoke (*M* = 553.62, *SD* = 182.16), replicating the results from Experiment 1. There was no significant main effect of testing vs. restudying or an interaction. Of greater interest are the significant differences in the number of words produced during the interviews. Consistent with Experiment 1, participants who wrote (*M* = 461.19, *SD* = 204.43) produced fewer words than those who spoke (*M* = 596.36, *SD* = 255.98). There was no significant main effect of testing vs. restudying or a significant interaction. Therefore, even though those who spoke produced more words, they were not producing more correct information. All *F-tests* and relevant descriptive statistics are reported in Table 6.

#### Phase 3

The Phase 3 data were checked for outliers, normality tests were conducted, and the relevant data were transformed, when necessary, via log transformations. The time to answer the questions was not recorded for Phase 3 because the questioning occurred via email. Multiple two-way ANOVAs and subsequent pairwise comparisons with Bonferroni corrections were conducted on the Phase 3 data to understand better the effects that the modality of questioning and testing vs. restudying had on memory report changes over time. These ANOVAs were conducted to compare (1) IBA, (2) OBA, (3) the proportion of true pointed questions answered correctly, (4) the proportion of false questions endorsed, (5) the proportion of false questions rejected, (6) open-ended question word counts, and (7) pointed-question word counts. These analyses all produced non-significant findings (*p*'s > 0.05). These findings suggest that the written superiority effect only extended to 1 week after encoding. All *F-tests* and relevant descriptive statistics are reported in Table 7.

**Table 7:** Experiment 2: Phase 3 F-tests and relevant descriptive statistics.

**Dependent variable**	**Statistical test**	**Descriptive statistics**	***95% C.I*.**
**IBA**			
Modality of questioning	*F*_(1, 39)_ = 2.864, *p* = 0.099, *$\eta_{p}^{2}$*= 0.068 (power = 0.41)	Written: *M* = 0.36, *SD* = 0.15 Spoken: *M* = 0.38, *SD* = 0.14	Written: 0.29–0.41 Spoken: 0.21–0.34
Test vs. Restudy	*F*_(1, 39)_ = 1.360, *p* = 0.251, *$\eta_{p}^{2}$*= 0.034 (power = 0.22)	Test: *M* = 0.35, *SD* = 0.15 Restudy: *M* = 0.29, *SD* = 0.14	Test: 0.28–0.40 Restudy: 0.22–0.36
Interaction	*F*_(1, 39)_ = 1.185, *p* = 0.283, *$\eta_{p}^{2}$*= 0.029 (power = 0.20)		
**OBA**			
Modality of questioning	*F*_(1, 38)_ = 0.605, *p* = 0.441, *$\eta_{p}^{2}$*= 0.016 (power = 0.13)	Written: *M* = 0.90, *SD* = 0.06 Spoken: *M* = 0.88, *SD* = 0.04	Written: 0.87–0.92 Spoken: 0.86–0.91
Test vs. Restudy	*F*_(1, 38)_ = 1.008, *p* = 0.322, *$\eta_{p}^{2}$*= 0.026 (power = 0.18)	Test: *M* = 0.88, *SD* = 0.05 Restudy: *M* = 0.90, *SD* = 0.05	Test: 0.86–0.90 Restudy: 0.87–0.92
Interaction	*F*_(1, 38)_ = 0.417, *p* = 0.522, *$\eta_{p}^{2}$*= 0.011 (power = 0.10)		
**True pointed questions**			
Modality of questioning	*F*_(1, 38)_ = 0.147, *p* = 0.704, *$\eta_{p}^{2}$*= 0.004 (power = 0.07)	Written: *M* = 0.83, *SD* = 0.09 Spoken: *M* = 0.82, *SD* = 0.11	Written: 0.79–0.87 Spoken: 0.77–0.86
Test vs. Restudy	*F*_(1, 38)_ = 0.945, *p* = 0.337, *$\eta_{p}^{2}$*= 0.024 (power = 0.17)	Test: *M* = 0.84, *SD* = 0.11 Restudy: *M* = 0.81, *SD* = 0.08	Test: 0.80–0.88 Restudy: 0.76–0.86
Interaction	*F*_(1, 38)_ = 0.721, *p* = 0.401, *$\eta_{p}^{2}$*= 0.019 (power = 0.14)		
**False questions endorsed**			
Modality of questioning	*F*_(1, 39)_ = 3.157, *p* = 0.083, *$\eta_{p}^{2}$*= 0.075 (power = 0.44)	Written: *M* = 0.47, *SD* = 0.31 Spoken: *M* = 0.30, *SD* = 0.29	Written: 0.34–0.60 Spoken: 0.16–0.44
Test vs. Restudy	*F*_(1, 39)_ = 0.175, *p* = 0.678, *$\eta_{p}^{2}$*= 0.004 (power = 0.07)	Test: *M* = 0.38, *SD* = 0.29 Restudy: *M* = 0.41, *SD* = 0.34	Test: 0.24–0.48 Restudy: 0.26–0.55
Interaction	*F*_(1, 39)_ = 0.050, *p* = 0.825, *$\eta_{p}^{2}$*= 0.001 (power = 0.05)		
**False questions rejected**			
Modality of questioning	*F*_(1, 39)_ = 0.403, *p* = 0.529, *$\eta_{p}^{2}$*= 0.010 (power = 0.10)	Written: *M* = 0.16, *SD* = 0.22 Spoken: *M* = 0.21, *SD* = 0.26	Written: 0.06–0.27 Spoken: 0.10–0.32
Test vs. Restudy	*F*_(1, 39)_ = 0.817, *p* = 0.372, *$\eta_{p}^{2}$*= 0.02 (power = 0.15)	Test: *M* = 0.15, *SD* = 0.20 Restudy: *M* = 0.22, *SD* = 0.28	Test: 0.05–0.25 Restudy: 0.11–0.33
Interaction	*F*_(1, 39)_ = 0.366, *p* = 0.549, *$\eta_{p}^{2}$*= 0.009 (power = 0.09)		
**Open-ended word counts**			
Modality of questioning	*F*_(1, 37)_ = 0.849, *p* = 0.363, *$\eta_{p}^{2}$*= 0.022 (power = 0.15)	Written: *M* = 306.18, *SD* = 160.30 Spoken: *M* = 253.21, *SD* = 114.51	Written: 235.17–356.05 Spoken: 190.57–320.09
Test vs. Restudy	*F*_(1, 37)_ = 1.047, *p* = 0.313, *$\eta_{p}^{2}$*= 0.028 (power = 0.18)	Test: *M* = 302.50, *SD* = 158.19 Restudy: *M* = 252.18, *SD* = 112.77	Test: 240.74–354.94 Restudy: 185.37–320.82
Interaction	*F*_(1, 37)_ = 2.681, *p* = 0.110, *$\eta_{p}^{2}$*= 0.068 (power = 0.39)		
**Pointed question word counts**			
Modality of questioning	*F*_(1, 37)_ = 0.113, *p* = 0.739, *$\eta_{p}^{2}$*= 0.003 (power = 0.06)	Written: *M* = 67.43, *SD* = 24.64 Spoken: *M* = 64.78, *SD* = 20.38	Written: 57.39–77.40 Spoken: 53.62–76.18
Test vs. Restudy	*F*_(1, 37)_ = 0.012, *p* = 0.915, *$\eta_{p}^{2}$*= 0.000	Test: *M* = 66.00, *SD* = 18.43 Restudy: *M* = 66.61, *SD* = 27.66	Test: 55.74–75.75 Restudy: 55.27–77.83
Interaction	*F*_(1, 37)_ = 0.035, *p* = 0.852, *$\eta_{p}^{2}$*= 0.001 (power = 0.05)		

All *F*-tests for each of the seven dependent variables are reported in the second column of the table as a function of the main effects for both the modality of questioning and testing vs. restudying, as well as the interactions. All descriptive statistics (means and standard deviations) have been included along with the 95% confidence interval.

#### Performance of participants who completed all sessions

It is possible that the number of participants that self-selected out at each phase could have impacted the previous findings. Therefore, two 2 × 2 × 2 mixed ANOVAs and subsequent pairwise comparisons with Bonferroni corrections were conducted for the IBAs and OBAs as a function of the modality of questioning and testing vs. restudying to assess potential differences across phases that may have been obscured by only examining participants who completed at least Phase 1 and Phase 2. Thus, the following analyses involve only participants who completed every phase (*N* = 43). There was a significant difference in IBA across phases, such that the IBAs lowered significantly from Phase 2 (*M* = 0.42, *SD* = *0.1*2) to Phase 3 (*M* = 0.32, *SD* = 0.15), consistent with Experiment 1. There were no other significant within-subject main effects or interactions or any significant testing vs. restudying between-subject main effects or interactions (*p*'s > 0.05). When examining OBAs, there were no significant between- or within-subject main effects or interactions. All *F-tests* and relevant descriptive statistics are reported in Table 8.

**Table 8:** Experiment 2: Performance of participants who completed all sessions.

**Dependent variable**	**Statistical test**	**Descriptive statistics**	***95% C.I*.**
**IBA**			
**Between-subject effects**			
Modality of questioning	*F*_(1, 39)_ = 4.432, *p* = 0.042, *$\eta_{p}^{2}$*= 0.102 (power = 0.58)		
Test vs. Restudy	*F*_(1, 39)_ = 0.526, *p* = 0.472, *$\eta_{p}^{2}$*= 0.013 (power = 0.11)		
Interaction	*F*_(1, 39)_ = 1.845, *p* = 0.182, *$\eta_{p}^{2}$*= 0.045 (power = 0.28)		
**Within-subject effects**			
Timing	*F*_(1, 39)_ = 19.554, *p* < 0.001, *$\eta_{p}^{2}$*= 0.334 (power = 0.99)	Phase 2: *M* = 0.42, *SD* = 0.12 Phase 3: *M* = 0.32, *SD* = 0.15	Phase 2: 0.38–0.45 Phase 3: 0.27–0.36
Timing^*^modality of questioning	*F*_(1, 39)_ = 0.020, *p* = 0.889, *$\eta_{p}^{2}$*= 0.001 (power = 0.05)	Phase 2^*^Written: *M* = 0.45, *SD* = 0.10 Phase 2^*^Spoken: *M* = 0.38, *SD* = 0.14 Phase 3^*^Written: *M* = 0.36, *SD* = 0.15 Phase 3^*^Spoken: *M* = 0.38, *SD* = 0.14	Phase 2^*^Written: 0.40–0.50 Phase 2^*^Spoken: 0.33–0.44 Phase 3^*^Written: 0.29–0.41 Phase 3^*^Spoken: 0.21–0.34
Timing^*^test vs. restudy	*F*_(1, 39)_ = 1.347, *p* = 0.253, *$\eta_{p}^{2}$*= 0.033 (power = 0.22)	Phase 2^*^Test: *M* = 0.42, *SD* = 0.14 Phase 2^*^Restudy: *M* = 0.42, *SD* = 0.10 Phase 3^*^Test: *M* = 0.35, *SD* = 0.15 Phase 3^*^Restudy: *M* = 0.29, *SD* = 0.14	Phase 2^*^Test: 0.36–0.46 Phase 2^*^Restudy: 0.36–0.47 Phase 3^*^Test: 0.28–0.40 Phase 3^*^Restudy: 0.22–0.36
Interaction	*F*_(1, 39)_ = 0.007, *p* = 0.933, *$\eta_{p}^{2}$*= 0.000		
**OBA**			
**Between-Subject Effects**			
Modality of Questioning	*F*_(1, 35)_ = 0.002, *p* = 0.962, *$\eta_{p}^{2}$*= 0.000		
Test vs. Restudy	*F*_(1, 35)_ = 0.212, *p* = 0.648, *$\eta_{p}^{2}$*= 0.006 (power = 0.08)		
Interaction	*F*_(1, 35)_ = 0.019, *p* = 0.891, *$\eta_{p}^{2}$*= 0.001 (power = 0.05)		
**Within-subject effects**			
Timing	*F*_(1, 35)_ = 0.372, *p* = 0.546, *$\eta_{p}^{2}$*= 0.011 (power = 0.10)	Phase 2: *M* = 0.89, *SD* = 0.06 Phase 3: *M* = 0.89, *SD* = 0.05	Phase 2: 0.87–0.90 Phase 3: 0.87–0.91
Timing^*^modality of questioning	*F*_(1, 35)_ = 1.785, *p* = 0.190, *$\eta_{p}^{2}$*= 0.049 (power = 0.28)	Phase 2^*^Written: *M* = 0.88, *SD* = 0.07 Phase 2^*^Spoken: *M* = 0.89, *SD* = 0.05 Phase 3^*^Written: *M* = 0.90, *SD* = 0.06 Phase 3^*^Spoken: *M* = 0.88, *SD* = 0.03	Phase 2^*^Written: 0.85–0.90 Phase 2^*^Spoken: 0.86–0.92 Phase 3^*^Written: 0.88–0.92 Phase 3^*^Spoken: 0.86–0.91
Timing^*^test vs. restudy	*F*_(1, 35)_ = 0.405, *p* = 0.528, *$\eta_{p}^{2}$*= 0.011 (power = 0.10)	Phase 2^*^Test: *M* = 0.88, *SD* = 0.07 Phase 2^*^Restudy: *M* = 0.88, *SD* = 0.04 Phase 3^*^Test: *M* = 0.89, *SD* = 0.05 Phase 3^*^Restudy: *M* = 0.90, *SD* = 0.05	Phase 2^*^Test:0.86–0.91 Phase 2^*^Restudy: 0.86–0.91 Phase 3^*^Test: 0.86–0.91 Phase 3^*^Restudy: 0.87–0.92
Interaction	*F*_(1, 35)_ = 0.714, *p* = 0.404, *$\eta_{p}^{2}$*= 0.020 (power = 0.14)		

All *F*-tests for each of the two dependent variables are reported in the second column of the table as a function of the main effects for the modality of questioning, testing vs. restudying, and timing (Phase 2 and Phase 3) as well as the interactions. All descriptive statistics (means and standard deviations) have been included along with the 95% confidence interval.

#### Rate of information loss

Multiple two-way ANOVAs and subsequent pairwise comparisons with Bonferroni corrections were conducted on the rate of information loss from Phase 1 to Phase 2 to Phase 3 for the immediately questioned groups and the rate of information loss from Phase 2 to Phase 3 for the restudy groups. There was a significant difference in the rate of information loss for IBA for participants in the immediately questioned groups [*F*_(5, 96)_ = 2.651, *p* = 0.027, $\eta_{p}^{2}$ = 0.121] (power = 0.99). There was a significant difference in IBA for those who wrote (*M* = 0.43, *SD* = 0.15) vs. spoke (*M* = 0.34, *SD* = 0.16); [*F*_(1, 96)_ = 8.594, *p* = 0.004, $\eta_{p}^{2}$ = 0.082] (power = 0.99). There was not a significant difference across Phase [*F*_(2, 96)_ = 2.504, *p* = 0.087, $\eta_{p}^{2}$ = 0.05]. Additionally, there was not a significant interaction between Phase and the immediately questioned groups (Written or Spoken) [*F*_(2, 96)_ = 0.276, *p* = 0.087, $\eta_{p}^{2}$ = 0.006]. There was not a significant difference in the rate of information loss for OBA for participants immediately questioned across the three phases: *F*_(5, 89)_ = 1.281, *p* = 0.279, $\eta_{p}^{2}$ = 0.067.

**Figure 4:**
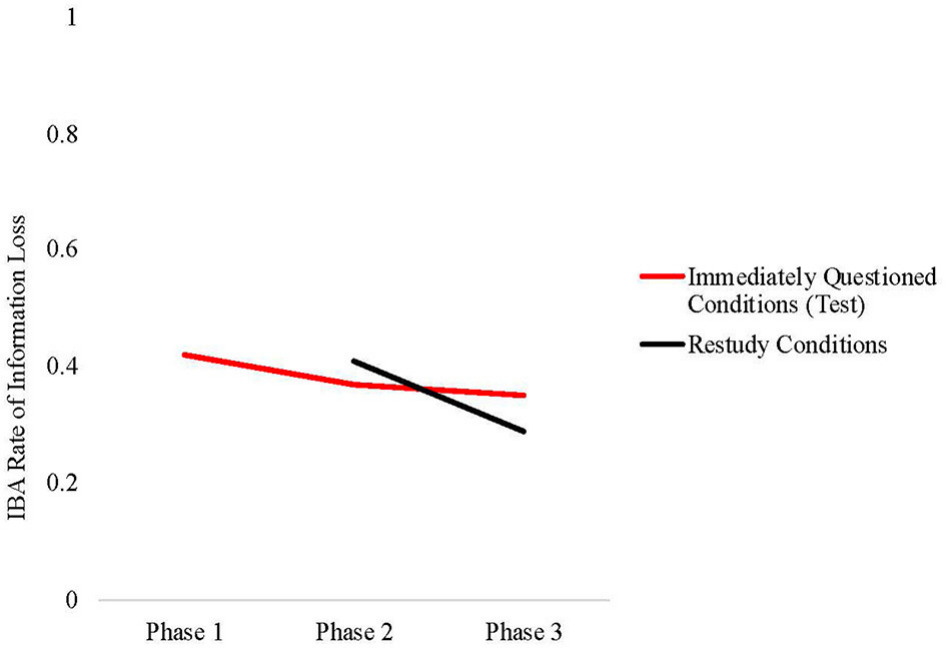
Experiment 2: Rate of information loss for IBA as a function of phase and test vs. restudy. The rate of information loss for IBA is presented along the y-axis as a function of each phase and whether a participant was immediately questioned or rewatched the video as a function of time (Phase). Over time, IBA decreases. Phase 2 occurs 1 week after Phase 1, and Phase 3 occurs 1 month after Phase 1.

For the restudy groups, there was a significant difference in the rate of information loss for IBA [*F*_(3, 51)_ = 5.035, *p* = 0.004, $\eta_{p}^{2}$ = 0.228] (power = 0.99), such that participants had a significantly lower IBA in Phase 3 (*M* = 0.29, *SD* = 0.14) compared to Phase 2 (*M* = 0.41, *SD* = 0.14); [*F*_(1, 51)_ = 10.752, *p* = 0.002, $\eta_{p}^{2}$ = 0.174] (power = 0.99). These findings can be seen in Figure 4. There was not a significant difference as a function of restudy (Written or Verbal): *F*_(1, 51)_ = 2.631, *p* = 0.111, $\eta_{p}^{2}$ = 0.049. Additionally, there was not a significant interaction: *F*_(1, 51)_ = 0.868, *p* = 0.356, $\eta_{p}^{2}$ = 0.017. Lastly, there was no significant difference in the rate of information loss for OBA: *F*_(3, 50)_ = 1.059, *p* = 0.375, $\eta_{p}^{2}$ = 0.060.

### Discussion

Though we replicated the Phase 2 IBA findings from Experiment 1, the present study did not otherwise statistically replicate the written superiority effect found in Experiment 1. It is possible that we did not find a written benefit because by removing the presence of an interviewer we reduced the chances for rapport building between the interviewer and interviewee. However, it is important to note that we also have lower statistical power in Experiment 2 than in Experiment 1. Previous research has been shown to facilitate memory reporting (Nash et al., 2014).

Given the similarity in design between Experiments 1 and 2, we also analyzed the data jointly. Additionally, because of the reduction in power in Experiment 2 due to a smaller sample size, we decided to combine the analyses to help increase power. We conducted a *post-hoc* power analysis and determined that combining the analyses provided us with a power estimate of 0.66. Additionally, the pandemic may have induced elevated levels of anxiety that could have reduced overall performance in Experiment 2, potentially masking or blunting experimental effects. Thus, collapsing across analyses allows us to assess additional relevant comparisons.

## Combined results

### Phase 1

In Phase 1, there was a main effect of the modality of questioning, such that participants in the Written-Written condition (*M* = 0.51, *SD* = 0.13) had a greater IBA proportion than those participants in the Spoken-Spoken condition (*M* = 0.42, *SD* = 0.14). There was also a main effect of questioning format, such that participants who completed Phase 1 in-person (*M* = 0.51, *SD* = 0.14) had a greater IBA proportion than the participants who completed Phase 1 virtually (*M* = 0.41, *SD* = 0.13). There was not a significant interaction. These findings provide support for the hypothesis that participants who write perform better than participants who speak. These findings are also suggestive of the benefit of questioning individuals in person. All *F-tests* and relevant descriptive statistics are reported in Table 9.

**Table 9:** Combined results: F-tests and relevant descriptive statistics.

**Dependent variable**	**Statistical test**	**Descriptive statistics**	***95% C.I*.**
**Phase 1**			
**IBA**			
Modality of questioning	*F*_(1, 76)_ = 9.662, *p* = 0.003, *$\eta_{p}^{2}$* = 0.113 (power = 0.88),	Written: *M* = 0.51, *SD* = 0.13 Spoken: *M* = 0.42, *SD* = 0.14	Written: 0.48–0.54 Spoken: 0.38–0.45
In-person vs. virtual	*F*_(1, 76)_ = 13.695, *p* < 0.001, *$\eta_{p}^{2}$* = 0.153 (power = 0.96)	In-Person: *M* = 0.51, *SD* = 0.14 Virtual: *M* = 0.41, *SD* = 0.13	In-Person: 0.48–0.54 Virtual: 0.38–0.44
Interaction	*F*_(1, 76)_ = 0.543, *p* = 0.464, *$\eta_{p}^{2}$*= 0.007 (power = 0.11)		
**Phase 2**			
**IBA**			
Modality of questioning	*F*_(3, 159)_ = 5.437, *p* = 0.001, *$\eta_{p}^{2}$* = 0.093 (power = 0.98)	Written-Written: *M* = 0.47, *SD* = 0.14 Spoken-Spoken: *M* = 0.37, *SD* = 0.12 Restudy-Written: *M* = 0.45, *SD* = 0.11	Written-Written: 0.45–0.49 Spoken-Spoken: 0.35–0.39 Restudy-Written: 0.43–0.47
In-person vs. virtual	*F*_(1, 159)_ = 8.213, *p* = 0.005, *$\eta_{p}^{2}$* = 0.049) (power = 0.82),	In-Person: *M* = 0.45, *SD* = 0.13 Virtual: *M* = 0.39, *SD* = 0.13	In-Person: 0.43–0.47 Virtual: 0.37–0.41
Interaction	*F*_(3, 159)_ = 2.038, *p* = 0.111, *$\eta_{p}^{2}$* = 0.037 (power = 0.70)		
**False questions endorsed**			
Modality of questioning	*F*_(3, 159)_ = 4.106, *p* = 0.008, *$\eta_{p}^{2}$* = 0.072) (power = 0.94)	Written-Written: *M* = 0.37, *SD* = 0.26 Restudy-Spoken: *M* = 0.17, *SD* = 0.23	Written-Written: 0.33–0.41 Restudy-Spoken: 0.13–0.21
In-person vs. virtual	*F*_(1, 159)_ = 0.002, *p* = 0.962, *$\eta_{p}^{2}$* = 0.000		
Interaction	*F*_(3, 159)_ = 0.194, *p* = 0.901, *$\eta_{p}^{2}$* = 0.004 (power = 0.13)		
**False questions rejected**			
Modality of questioning	*F*_(3, 159)_ = 4.918, *p* = 0.003, *$\eta_{p}^{2}$* = 0.085) (power = 0.97)	Written-Written: *M* = 0.21, *SD* = 0.24 Restudy-Written: *M* = 0.36, *SD* = 0.27 Spoken-Spoken: *M* = 0.15, *SD* = 0.19	Written-Written: 0.17–0.25 Restudy-Written: 0.32–0.40 Spoken-Spoken: 0.12–0.18
In-person vs. virtual	*F*_(1, 159)_ = 2.069, *p* = 0.152, *$\eta_{p}^{2}$* = 0.013 (power = 0.30)		
Interaction	*F*_(3, 159)_ = 0.796, *p* = 0.498, *$\eta_{p}^{2}$* = 0.015 (power = 0.34)		
**Phase 3**			
**IBA**			
Modality of questioning	*F*_(3, 103)_ = 2.335, *p* = 0.078, *$\eta_{p}^{2}$* = 0.064) (power = 0.76)		
In-person vs. virtual	*F*_(1, 103)_ = 5.381, *p* = 0.022, *$\eta_{p}^{2}$* = 0.050) (power = 0.64)	In-Person: *M* = 0.39, *SD* = 0.13 Virtual: *M* = 0.33, *SD* = 0.12	In-Person: 0.37–0.42 Virtual: 0.31–0.35
Interaction	*F*_(3, 103)_ = 0.467, *p* = 0.706, *$\eta_{p}^{2}$* = 0.013 (power = 0.21)		

All *F*-tests for each dependent variable are reported in the second column of the table as a function of the main effects for both the modality of questioning and in-person vs. virtual interview format, as well as the interactions. Each of the three phases has been included here. All descriptive statistics (means and standard deviations) have been included along with the 95% confidence interval.

### Phase 2

In Phase 2, there was a significant main effect of the modality of questioning, such that participants in the Written-Written (*M* = 0.47, *SD* = 0.14) condition had a greater IBA than those in the Spoken-Spoken (*M* = 0.37, *SD* = 0.12) condition. Participants in the Restudy-Written (*M* = 0.45, *SD* = 0.11) condition also had a greater IBA than those in the Spoken-Spoken condition. There was also a significant main effect of question format, such that participants who completed Phase 2 in person (*M* = 0.45, *SD* = 0.13) had a greater IBA than those who completed Phase 2 virtually (*M* = 0.39, *SD* = 0.13). There was no significant interaction between the modality of questioning and question format. These findings again provide support for the hypothesis that participants who write perform better than participants who speak, and this effect remains in Phase 2 (1 week later). Also of interest is the lack of a traditional testing effect. One would expect to find a testing benefit for those who are questioned immediately (Written-Written and Spoken-Spoken) compared to the restudy conditions who are not questioned until a week later (Restudy-Written and Restudy-Spoken). We found that writing overcomes the anticipated benefit of testing as indicated by better performance in the Restudy-Written and Written-Written conditions compared to those in the Spoken-Spoken condition. Lastly, these findings are suggestive of the benefit of questioning participants in person compared to remotely, consistent with findings from Phase 1. All *F-tests* and relevant descriptive statistics are reported in Table 9.

There was also a significant main effect of the modality of questioning on the proportion of false questions endorsed such that participants in the Written-Written (*M* = 0.37, *SD* = 0.26) condition incorrectly endorsed the false questions at a higher rate than those in the Restudy-Spoken (*M* = 0.17, *SD* = 0.23) condition. There was no significant main effect of question format or interaction. Restudy-Spoken participants were less likely to endorse a false question, which is consistent with research supporting that studying (as opposed to testing) promotes more verbatim processing (Reyna and Brainerd, 1995). This also could explain the improved ability to answer those questions for those who studied the video twice (i.e., Restudy-Spoken). Interestingly, despite writing resulting in better performance for open-ended questioning, when asked pointed questions, Written-Written participants endorsed more false questions than those who spoke. All *F-tests* and relevant descriptive statistics are reported in Table 9.

Lastly, there was a significant main effect of the modality of questioning on the proportion of false questions correctly rejected, such that participants in the Restudy-Written condition (*M* = 0.36, *SD* = 0.27) rejected the false questions more often than participants in the Spoken-Spoken (*M* = 0.15, *SD* = 0.19) and Written-Written (*M* = 0.21, *SD* = 0.24) conditions. There was not a significant main effect of question format or interaction. These findings are also consistent with the literature that suggests that studying promotes verbatim processing because those who restudied the video (Restudy-Written) were better able to reject false questions. All *F-tests* and relevant descriptive statistics are reported in Table 9.

### Phase 3

In Phase 3, there was a significant main effect of the question format, such that participants who had completed Phases 1 and 2 in person (*M* = 0.39, *SD* = 0.13) had a greater IBA in Phase 3 than those who completed Phases 1 and 2 virtually (*M* = 0.33, *SD* = 0.12). There was a marginally significant main effect of the modality of questioning, but no significant interaction. These findings provide marginal support for the benefits of questioning participants in person rather than virtually, even over an extended period. Lastly, any differential effects of the modality of questioning seen during Phases 1 and 2 diminished by Phase 3. All *F-tests* and relevant descriptive statistics are reported in Table 9.

## General discussion

Having individuals provide either a written or spoken memory report differentially impacts both the type and the number of details reported. The findings from Experiment 1 are consistent with research supporting a written superiority effect, which is still present 1 week later (Kraus et al., 2017; Sauerland et al., 2014). Consistent with previous research, speaking is more productive, although not as efficient: Participants who wrote had longer completion times but equivalent word counts. Additionally, we found that the written advantage diminishes by Phase 3. These findings may be indicative of a shift from verbatim to gist recall; the fuzzy-trace theory literature suggests that detailed (verbatim) memories are forgotten more quickly than gist memories (Ahmad et al., 2017). However, the attrition rates from Phase 2 to Phase 3 in both experiments are important to consider. Mainly, did the drop off in response rate occur randomly, or was it due to those with poorer memories choosing not to respond to the Phase 3 email? However, in Phases 1 and 2, memory performance was not significantly different between those who did and did not complete the Phase 3 email for Experiment 1 (*p* = 0.26) or Experiment 2 (*p* = *0.2*9). Therefore, the attrition rates among the four conditions were likely random.

Additionally, for the pointed questions, watching the video twice and then providing a report (Restudy-Written and Restudy-Spoken) appears to be more beneficial than watching and then providing a report (Written-Written and Spoken-Spoken). In Phase 2, the lack of a testing effect is seen between the Restudy-Spoken and the Written-Written condition and the Restudy-Written and Spoken-Spoken condition. These findings are consistent with research supporting the idea that restudying promotes verbatim processing (Reyna and Brainerd, 1995). By watching the video twice, the restudy conditions allowed participants to manage the false questions more appropriately by either endorsing them less often or correctly rejecting them more often. By watching the video twice, these participants had an opportunity to update their memories and fill in gaps from watching the video the first time, whereas the immediately questioned individuals (Written-Written and Spoken-Spoken) did not have this opportunity.

However, the nature of the memory tests is important to consider. In an eyewitness setting, a witness is likely interviewed but may not anticipate the interview's questions or structure. This is not necessarily the case in a classroom setting, aside from pop quizzes, as students typically know what they need to study and the type of testing format they will engage in. Therefore, the surprising nature of questioning/testing in an eyewitness setting compared to a classroom setting could impact individuals' expectations and, subsequently, the demands placed on their memories. Therefore, the intention to remember information could affect the details an individual remembers. Thus, the present findings may not generalize to these different testing scenarios.

Experiment 2 removed some of the social components inherent to a traditional interview. Previous research suggests that an interviewer can have positive and negative effects on a witness (Bergmann et al., 2004). We only replicated findings in Phase 2 for IBA; therefore, it is possible that positive and negative effects of interviewer presence balanced out to mostly produce null effects.

Interestingly, writing was beneficial for the open-ended questions but not for the pointed questions. Questions that promote open-ended responses allow individuals to benefit from the self-monitoring advantages associated with writing, a benefit largely eliminated by pointed questions. Also, answering open-ended questions allows self-pacing to play a greater role than when answering pointed questions. Additionally, including false pointed questions could have made it difficult for the benefits of writing to be revealed. Whereas with open-ended questions, participants have the flexibility to choose what they report, this was not an option for the pointed questions as a response was required. Future research will be needed to evaluate these ideas.

Additionally, it is important to consider the impact of the COVID-19 pandemic, which brought with it adverse factors beyond just a move to remote questioning. Research has shown that a large majority of the population experienced one or more of the following mental health problems during the pandemic; stress, anxiety, and/or depression (Shah et al., 2021). Additionally, extended periods of quarantining have been associated with increased mental health problems, and during the pandemic, research has shown that undergraduate students showed elevated levels of these symptoms while quarantining (Hamaideh et al., 2022; Shah et al., 2021). Mental health problems can impact day-to-day functioning, and given the differential effects of stress on memory (Schwabe et al., 2012), it is possible that Experiment 2 performance was affected more by the pandemic than by virtual questioning via Zoom. Therefore, future research is needed to determine if virtual questioning really is worse than in-person questioning.

### Limitations

A limitation of the present research is that even though the researchers were trained to be systematic in their responses to each question, it is likely that participants who spoke were more likely to look at and/or engage with the researcher than participants who wrote. When speaking, it is more natural to engage with the other individual; consequently, the speaking conditions (Restudy-Spoken and Spoken-Spoken) naturally induce more researcher interactions than the written conditions (Restudy-Written and Written-Written). This may explain why participants in the spoken conditions were more likely to report that they did not know an answer rather than providing a specific answer to the pointed questions for fear of being judged by the research assistant. Also, it is possible that, even with training, the researchers may have inadvertently cued the participants that their responses were correct or incorrect, making it possible that accidental confirmatory feedback played a role in the present study's findings (Zaragoza et al., 2001). Researchers that had never watched the video would have alleviated this concern.

Another concern of this study is that in Phase 3 (conducted by email), the modality of questioning changed for the speaking conditions (Restudy-Spoken and Spoken-Spoken) but not for the written conditions (Restudy-Written and Written-Written). Given the confounding nature of this change, caution should be used when interpreting the Phase 3 data. However, it is important to note that some of the effects persisted in Phase 3, which indicates that memories are different such that the present findings are not just a reflection of the modality of questioning but rather evidence that it disentangles prior modality of testing from the current modality of testing. Further work should be done to elucidate these mechanisms. Additionally, because Phase 3 occurred via email, motivation to perform could have been impacted during the memory test as no research assistant was present to monitor this phase's completion. Though participants were allowed to take as much time as needed to respond, they may have taken breaks or multi-tasked while completing the questionnaire, which did not happen in Phases 1 and 2. Having participants complete Phase 3 (in person or virtually) with a research assistant present would have alleviated some of these concerns.

Another limitation of the current study is that the video depicted events at an all-boys summer camp, and the events portrayed are emotionally neutral. Thus, the present findings may not generalize to more realistic scenarios. Research suggests that stress can negatively impact memory (Christianson, 1992); in the context of eyewitnesses, for example, events are likely to be stress-inducing or emotionally charged. Thus, it is possible that the written superiority effect seen in Phases 1 and 2 may not withstand more life-like, stressful events. This may also explain why the findings from Experiment 1 mostly did not replicate in Experiment 2. Previous research support for virtual-associated recall advantages may be contingent on the reporting of emotional events; the removal of social factors in the present instance may be unlikely to provide a benefit to memory reports. When recalling a neutral event, individuals may not find it important to report all critical pieces of information (in fact, it may not be clear what is a critical piece of information). Event relevance likely induces greater levels of engagement because participants feel that it is important to contribute to the interview. Utilizing a more memorable stimulus video may be helpful to determine if the written superiority effect can extend to 1 month (or greater) following initial encoding; a 1-month retention interval may be too long for the non-memorable event we used.

### Future directions

More research is needed to elucidate how the interviewer's presence impacts memory reports and how that may interact with the modality of questioning. It is important to find ways to conduct interviews without an interviewer being present because it will allow educators, legal actors, medical professionals, and others to better delegate their already limited resources. For example, in a case involving multiple witnesses, a precinct must work fast and efficiently to obtain the most detailed and accurate reports. Therefore, if precincts could question witnesses remotely, this would greatly reduce the workload. Moreover, it is important to determine whether it is better to conduct this questioning by speaking to a witness or asking them to write down what they remember.

Future research should investigate the written superiority effect as a function of different written methods. Past research reveals that varied written questioning structures can differentially influence the occurrence of a written superiority effect (Kraus et al., 2017). Though the SAI worked in applied settings, investigating how free-recall and semi-structured questioning (or pointed question) formats impact the presence of a written superiority effect is also important. If self-monitoring is a reason for the written superiority effect, then this is a key next step. The present study provides some support for this idea as the written superiority effect seen here partly depended on the type of question asked. More specifically, writing tends to be more beneficial for open-ended compared to pointed questions.

Additionally, it is important to consider the retention interval used here and how future research studies should implement a design that includes a group of participants who did not get tested in Phase 2. By including this additional condition, researchers would gain a greater understanding of the impact of modality on retention, particularly on long-term retention intervals. Here, the Phase 3 test always comes before the Phase 2 test, so more research is needed to assess actual study retention interval effects.

There are a multitude of ways in which interviews can be conducted. Accordingly, it is of interest to identify practices that will help obtain the most accurate memory reports, whether in the classroom, at a crime scene, or in a medical office. This study allowed us to take a step toward identifying some of these factors, specifically the modality of questioning and timing, and how these factors can elicit the highest quality memory reports. To start, consistent with our first hypothesis prediction, the current study found that writing leads to better quality memory reports compared to speaking, and the benefit is present 1 week later. Interestingly, we also predicted a traditional testing effect. However, we found that writing mitigates this anticipated testing benefit, though this depended on whether a pointed or open-ended question was asked. Additionally, restudying (vs. immediate testing) led to better performance for the false pointed questions. However, the better performance operated differently depending on whether participants wrote or spoke following restudying.

Therefore, we can conclude that the influence of the modality of questioning on memory reports is susceptible in several ways to the types of questions asked, which suggests that one modality may be more suited for different domains. Or that implementing a combination of modalities can produce more accurate memory reports as a function of the types of questions asked. Whether in a classroom, at a doctor's office, or at a crime scene, one could feasibly be questioned using an assortment of open-ended and pointed questions and subsequently asked to respond verbally or with a written statement. Therefore, continuing to elucidate the contexts in which the modality of questioning, timing, and the types of questions asked work to improve overall memory reporting will be insightful and relevant to the global understanding of the mechanisms of memory.

## Data Availability

The datasets presented in this study can be found in online repositories. The names of the repository/repositories and accession number(s) can be found below: https://osf.io/4v3z7/.
